# Negative cooperativity in the formation of H-bond networks involving primary anilines†

**DOI:** 10.1039/d4sc03719g

**Published:** 2024-07-03

**Authors:** Fergal E. Hanna, Alexander J. Root, Markus Schade, Christopher A. Hunter

**Affiliations:** a Yusuf Hamied Department of Chemistry, University of Cambridge Lensfield Road Cambridge CB2 1EW UK herchlsmith.orgchem@ch.cam.ac.uk; b Chemistry, Oncology R&D, AstraZeneca 1 Francis Crick Avenue Cambridge CB2 0AA UK

## Abstract

Networks of H-bonds can show non-additive behaviour, where the strength of one interaction perturbs another. The magnitude of such cooperative effects can be quantified by measuring the effect of the presence of an intramolecular H-bond at one site on a molecule on the association constant for formation of an intermolecular H-bond at another site. This approach has been used to quantify the cooperativity associated with the interaction of a primary amine with two H-bond acceptors. A series of compounds that have an intramolecular H-bond between an aniline NH_2_ group and a pyridine nitrogen were prepared, using polarising substituents on the pyridine ring to vary the strength of the intramolecular H-bond. The presence of the intramolecular interaction was confirmed by X-ray crystallography in the solid state and NMR spectroscopy in *n*-octane solution. UV-vis absorption titrations were used to measure the association constants for formation of an intermolecular H-bond with tri-*n*-butyl phosphine oxide in *n*-octane. Electron-donating substituents on the pyridine ring, which increase the strength of the intramolecular H-bond, were found to decrease the strength of the intermolecular H-bond between the aniline and the phosphine oxide. The results were used to determine the H-bond donor parameters for the anilines, *α*, and there is a linear relationship between the values of *α* and the H-bond acceptor parameter of the pyridine group involved in the intramolecular H-bond, *β*. The slope of this relationship was used to determine the cooperativity parameter (*κ* = −0.10), which quantifies the negative allosteric cooperativity between the two H-bonding interactions. Calculated molecular electrostatic potential surfaces of the anilines quantitatively reproduce the experimental result, which suggests that effects are electrostatic in origin, either due to polarisation of the NH bonds or due to secondary electrostatic interactions between the two H-bond acceptors.

## Abstract 2

Primary anilines have two H-bond donor sites that are coupled, and as the strength of the H-bond interaction at one site increases, the strength of the interaction at the other site decreases.

## Graphical abstract

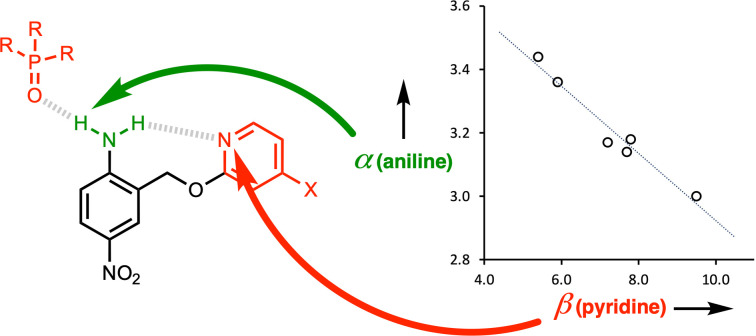

## Introduction

**Fig. 1:**
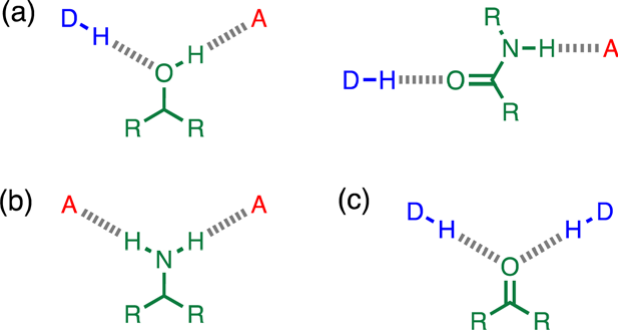
Cooperative H-bond networks. (a) Interaction of an alcohol or an amide with a H-bond donor (DH) and a H-bond acceptor (A). (b) Interaction of a primary amine with two H-bond acceptors. (c) Interaction of a carbonyl oxygen with two H-bond donors.

Non-covalent interactions play a pivotal role in determining the structure and properties of materials,^1^ biomolecules^2^ and supramolecular systems.^3^ The thermodynamic properties of a non-covalent interaction can be predicted using parameters that describe the individual functional groups involved.^4^ However in assemblies that involve multiple polar interactions, cooperative effects can make a significant contribution to the overall stability.^5^ Positive allosteric cooperativity in multiply H-bonded complexes of alcohols^6,7^ and amides^8,9^ has been studied using calorimetry,^10–12^ NMR,^6,13–15^ and IR spectroscopy,^16–19^ and computational methods suggest that bond polarisation plays a major role in these cooperative phenomena.^20–27^ Formation of a H-bond to the H-bond acceptor increases the polarity of the H-bond donor and *vice versa*. We have quantified these cooperative effects in amides and phenols by measuring the relationship between the strength of an intramolecular H-bond on the strength of a second intermolecular H-bond made with the same functional group.^6–9^ These experiments were used to measure cooperativity between interactions involving a H-bond donor and a H-bond acceptor (Fig. 1a), which leads to positive cooperativity. However, there are other classes of H-bond network, where the cooperative effects may be quite different. For example, Fig. 1b shows the interaction of a primary amine with two H-bond acceptors, and Fig. 1c shows the interaction of a carbonyl oxygen with two H-bond donors. Here we describe experiments to measure cooperativity between interactions of a primary aniline NH_2_ group with two different H-bond acceptors in *n*-octane solution. We show that there is negative allosteric cooperativity in these networks and that the magnitude of the effect depends on the polarity of the H-bond acceptors.

**Fig. 2:**
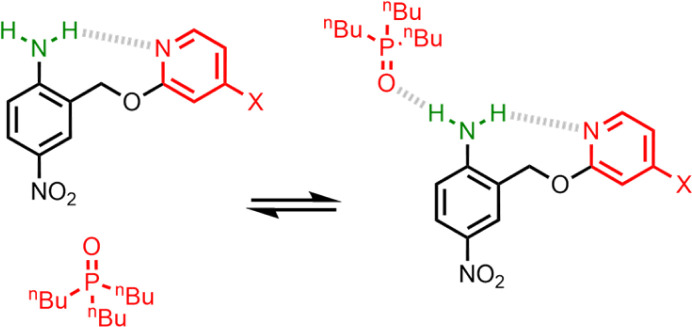
Interaction of a H-bonded aniline (green) with a phosphine oxide. X is a substituent that modulates the H-bond acceptor properties of the pyridine (red).

The interaction of primary aromatic amines with H-bond acceptors has been studied using IR spectroscopy to estimate the enthalpy of the first and second H-bond formed with the two NH groups.^28–31^ In the majority of cases, the second H-bond was found to be weaker than the first, which is consistent with measurements of the enthalpy of solution of anilines.^32^ IR spectroscopy was used to study formation of intermolecular H-bonds with primary anilines that are involved in an intramolecular H-bond, and changes in the NH stretching frequency suggest that the intermolecular H-bond leads to a weakening of the intramolecular H-bond.^33^ Here we quantify the effects of negative allosteric cooperativity on the free energy changes associated with H-bonding interactions with anilines. Fig. 2 illustrates the approach. We have shown previously that the architecture in Fig. 2 promotes an intramolecular H-bond between the pyridine and aniline NH_2_ group.^9^ The strength of this intramolecular interaction can be modulated by varying the X substituent, which changes the H-bond acceptor properties of the pyridine nitrogen, and the methylene spacer minimises any through bond communication between the pyridine and aniline units. Measurement of the association constant for formation an intermolecular H-bond with tri-*n*-butyl phosphine oxide as a function of the X substituent therefore provides a method for quantifying the effect of the strength of the intramolecular H-bond on the strength of the intermolecular H-bond.

## Results and discussion

**Fig. 3:**
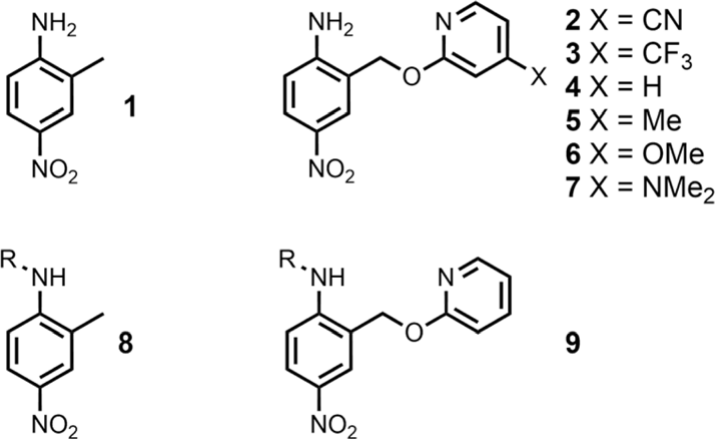
Chemical structures of anilines 1–9. R = *n*-hexyl.

The experiment in Fig. 2 requires a set of anilines equipped with different pyridine derivatives (Fig. 3). Compound 1 is commercially available and provides an aniline that does not have an intramolecular H-bond as a reference point. Compounds 3–7 were prepared as described previously, and compound 2 was synthesised using a similar route (see ESI† for details).^9^ Secondary anilines 8 and 9 were used to investigate whether intermolecular H-bonding with tri-*n*-butyl phosphine oxide would compete with the intramolecular pyridine-aniline H-bond. These compounds were prepared by alkylation of 1 and 4 using sodium hydride and 1-bromohexane (see ESI†).

### Intramolecular H-bonding

**Fig. 4:**
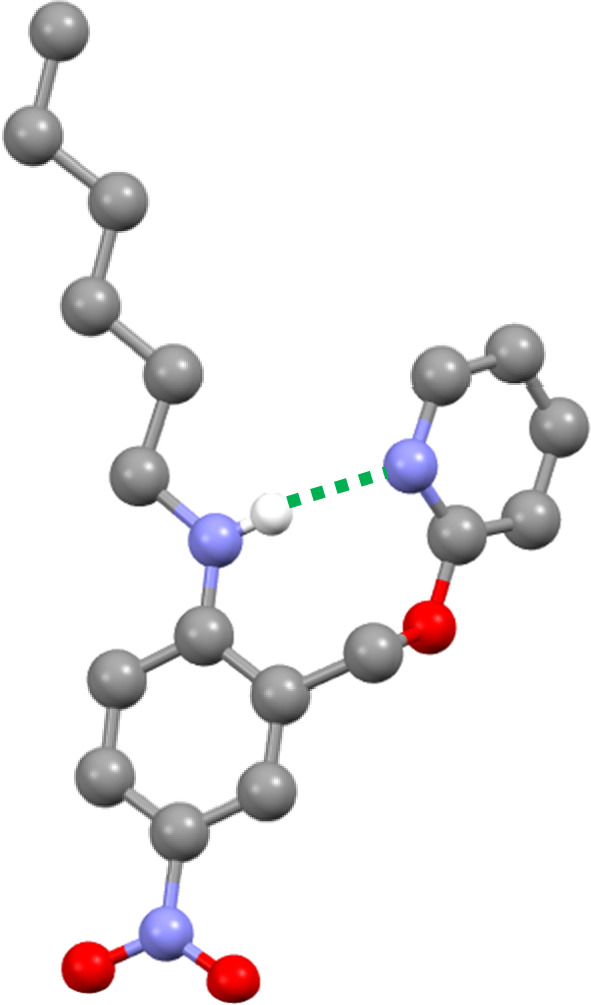
Molecular structure of 9 taken from the X-ray crystal structure. The intramolecular H-bond is shown as a dotted green line.

The three-dimensional structure of compound 9 was determined by single crystal X-ray diffraction, and the intramolecular H-bond illustrated in Fig. 2 is clearly present (Fig. 4). The ^1^H NMR spectrum in *n*-octane indicates that this interaction is also present in solution: the chemical shift of the signal due to the NH proton of 9 is 6.12 ppm, a 2.3 ppm downfield shift compared with the corresponding signal in compound 8 (3.84 ppm), which does not have a pyridine group that can form an intramolecular H-bond. Dilution experiments showed no change in chemical shift with concentration for either compound, which confirms that the downfield shift in 9 is due to an intramolecular interaction rather than aggregation (see ESI†).

The ^1^H NMR spectra of compounds 1–7 in *n*-octane suggest that there is an intramolecular H-bond between the pyridine and aniline NH_2_ group in all of compounds 2–7 (Table 1): the chemical shift of the signal due to the NH_2_ group shows a downfield shift of 0.8–1.6 ppm relative to compound 1. In the primary anilines, the signal due to the NH_2_ group is the fast exchange average of the signals due to the H-bonded and non-H-bonded protons, so the size of the downfield shift is about half the value observed for the secondary aniline 9, where the NH proton is always H-bonded. Table 1 shows that the size of the downfield shift observed for the NH_2_ signal depends on the X substituent. The largest chemical shifts were observed for the most electron-donating X substituents, and the values correlate with the H-bond acceptor parameters (*β*) of the corresponding 4-X-pyridines (*R*^2^ = 0.99).^9^ These observations indicate that the properties of the intramolecular H-bond in compounds 2–7 depend on the H-bond acceptor properties of the pyridine nitrogen. Further confirmation of the presence of the intramolecular H-bond was found in the ^1^H–^15^N HMBC spectrum of 4 in *n*-octane, where a cross-peak was observed between the ^15^N signal due to the pyridine nitrogen and ^1^H signal due to the aniline NH_2_ group (see Fig. S13†).

**Table 1:** ^1^H NMR chemical shifts of the signal due to the NH_2_ group of the primary anilines in *n*-octane at 298 K

Compound	X	*β*(pyridine)^a^	*δ*/ppm
1	—	—	3.84
2	CN	5.4	4.62
3	CF_3_	5.8	4.68
4	H	7.2	4.96
5	Me	7.7	5.06
6	OMe	7.8	5.16
7	NMe_2_	9.5	5.44

a H-Bond acceptor parameter (*β*) of the corresponding 4-X-pyridine from ref. 9.

### Intermolecular H-bonding

**Fig. 5:**
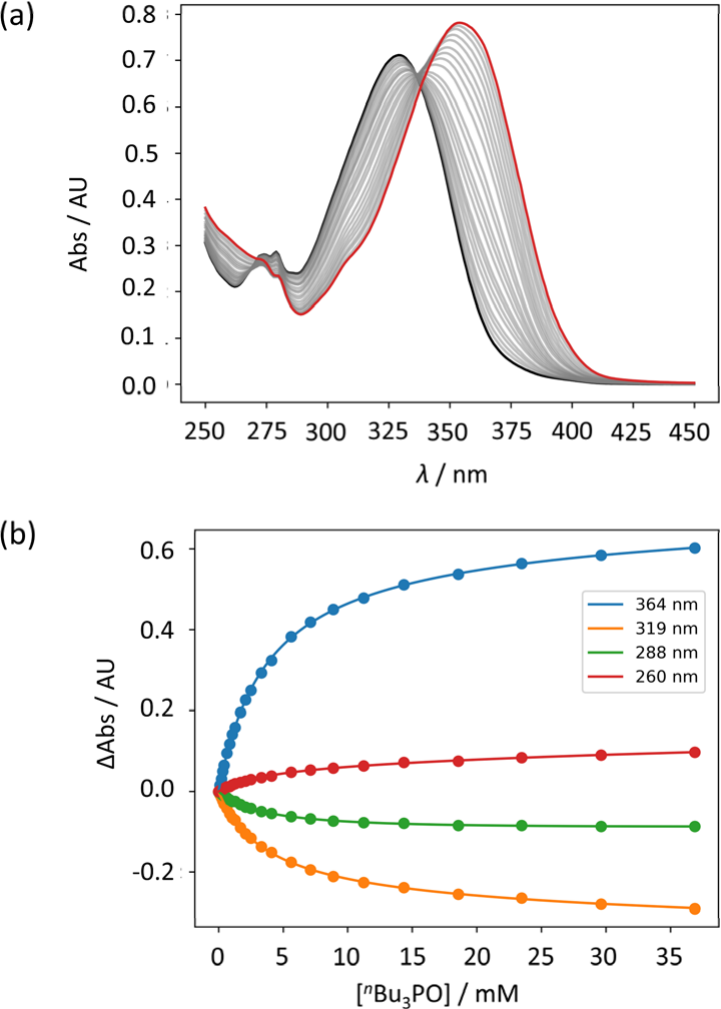
(a) UV-vis absorption spectra for the titration of tri-*n*-butyl phosphine oxide into 4 (50 μM in *n*-octane, at 298 K). The spectrum of the host 4 and the final point of the titration are reported in black and in red, respectively. (b) The lines show the best fit of the change in absorbance at four different wavelengths (points) to a 1:2 binding isotherm (*K*_1_ = 272 M^−1^, *K*_2_ = 7 M^−1^).

**Fig. 6:**
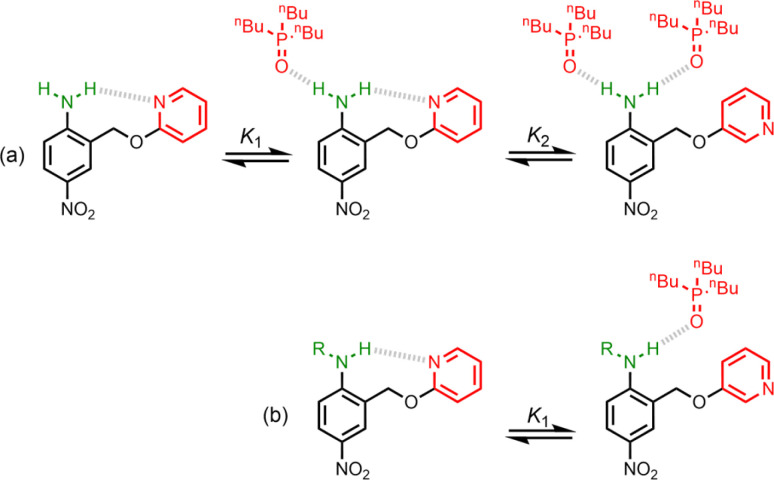
(a) Formation of a 1:2 complex between tri-*n*-butyl phosphine oxide and 4. (b) Formation of a 1:1 complex between tri-*n*-butyl phosphine oxide and 9. R = *n*-hexyl.

UV-vis titration experiments were carried out with anilines as the host and tri-*n*-butyl phosphine oxide as the guest in *n*-octane at 298 K. Fig. 5a shows an example of a titration with compound 4. On addition of the guest, the absorption band at 319 nm decreased, and a new band due to the H-bonded complex appeared at 364 nm. There is no well-defined isosbestic point in Fig. 5a, which indicates that this is not a simple two-state equilibrium, and the best fit to the experimental data was obtained using a 1:2 binding isotherm with a weak second binding interaction. It is possible that the second binding event involves breaking the intramolecular H-bond in 4 in order to make the 1:2 complex shown in Fig. 6a. To test this hypothesis, the interaction of tri-*n*-butyl phosphine oxide with secondary aniline 9 was investigated. In this case, formation of the 1:1 complex would require breaking of the intramolecular H-bond (Fig. 6b). The association constant for the 1:1 complex formed with 9 is *K*_1_ = 30 ± 6 M^−1^, which is an order magnitude lower than the value of *K*_1_ measured for 4, and the same order of magnitude as the value of *K*_2_. This result suggests that the intramolecular H-bond is intact in the 1:1 complex formed by 4 and tri-*n*-butyl phosphine oxide, and that formation of the 1:2 complex involves breaking of the intramolecular interaction.^34^

**Fig. 7:**
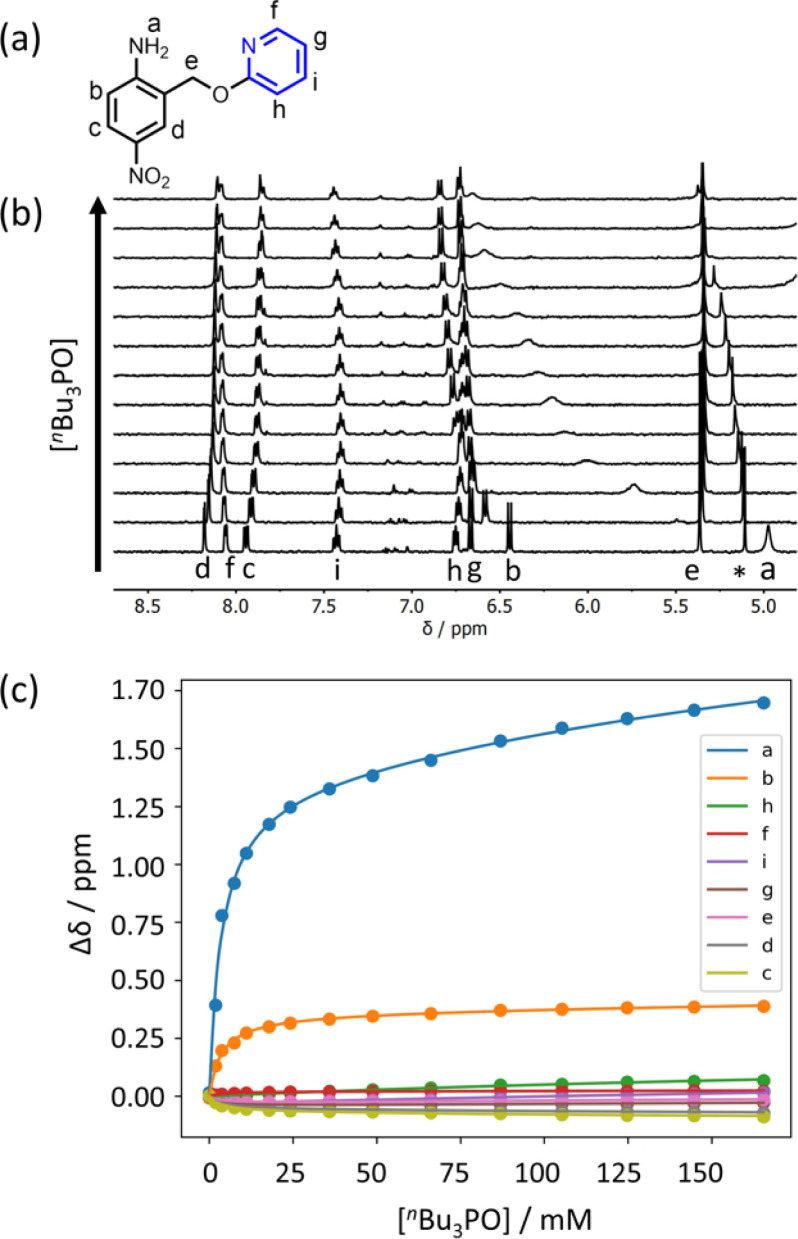
(a) Proton labelling scheme for compound 4. (b) Partial 400 MHz ^1^H NMR spectra for titration of tri-*n*-butyl phosphine oxide into 4 (0.31 mM in *n*-octane, at 298 K). An impurity in the solvent is labelled with an asterisk. (c) The lines show the best fit of the chemical shift data (points) to a 1:2 binding isotherm (*K*_1_ = 300 M^−1^, *K*_2_ = 3 M^−1^).

**Fig. 8:**
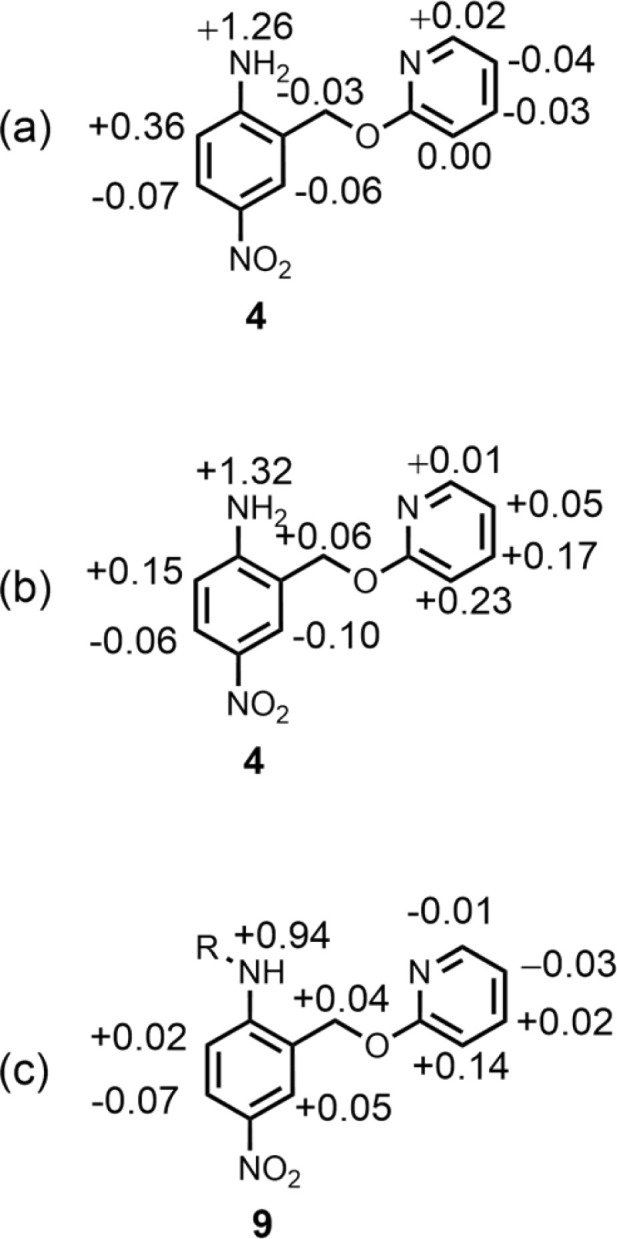
Limiting complexation-induced changes in ^1^H NMR chemical shift (ppm) in *n*-octane at 298 K. (a) Difference between the chemical shifts for free 4 and the 1:1 complex with tri-*n*-butyl phosphine oxide. (b) Difference between the chemical shifts for the 1:1 and 1:2 complexes formed with tri-*n*-butyl phosphine oxide. (c) Difference between the chemical shifts for free 9 and the 1:1 complex with tri-*n*-butyl phosphine oxide. R = *n*-hexyl.

Further insight into the structures of the complexes was obtained from ^1^H NMR spectroscopy. Fig. 7b shows ^1^H NMR spectra for titration of tri-*n*-butyl phosphine oxide into 4 in *n*-octane solution, and Fig. 7c shows the best fit of the data to a 1:2 binding isotherm. The value of *K*_1_ is close to the value measured by UV-vis absorption spectroscopy (Table 2). Fig. 8a and b show the limiting complexation-induced changes in chemical shift. The large increase in chemical shift observed for the signal due to the NH_2_ protons is indicative of H-bonding interactions with phosphine oxide in both the 1:1 and 1:2 complexes. In the 1:1 complex, the next largest change in chemical shift (+0.33 ppm) was observed for the aromatic proton *ortho* to the amino group (proton b in Fig. 7), which is consistent with a close contact with the phosphine oxide at this site, as suggested by the structure shown in Fig. 6a. In the 1:2 complex, the next largest change in chemical shift (+0.23 ppm) was observed for a proton on the pyridine ring (proton h in Fig. 7), which suggests that the pyridine ring flips over to allow close contact between this proton and the second phosphine oxide, consistent with breaking the intramolecular H-bond (see Fig. 6a). A ^1^H NMR titration of tri-*n*-butyl phosphine oxide into 9 was carried out for comparison, and the limiting complexation-induced changes in chemical shift for formation of the 1:1 complex are shown in Fig. 8c. A large positive change in chemical shift (+0.94 ppm) was observed for the NH proton, confirming that the large increase in chemical shift observed in the 1:2 complex of 4 is due to a H-bonding interaction with the phosphine oxide. The next largest change in chemical shift (+0.14 ppm) was again observed for proton h on the pyridine ring, which indicates that this proton does indeed report on a change in conformation associated with breaking the intramolecular H-bond (Fig. 6b). The NMR results therefore confirm the conclusion that the weak second binding event observed for the interaction of tri-*n*-butyl phosphine oxide with 4 is due to competition with the intramolecular H-bond, but that the intramolecular interaction is intact in the 1:1 complex.

**Table 2:** Association constants for formation of 1:1 complexes with tri-*n*-butyl phosphine oxide measured by UV-vis absorption titrations in *n*-octane at 298 K and corresponding H-bond parameters calculated using eqn (1)^a^

Compound	X	*β*(pyridine)^b^	*K*_1_/M^−1^	*α*(aniline)
1	—	—	340 ± 20	3.2
2	CN	5.4	820 ± 30	3.4
3	CF_3_	5.8	586 ± 14	3.4
4	H	7.2	272 ± 11	3.2
5	Me	7.7	240 ± 50	3.1
6	OMe	7.8	290 ± 20	3.2
7	NMe_2_	9.5	134 ± 3	3.0
8	—	—	59 ± 2	2.8
9	—	—	30 ± 6	—c

a Errors are quoted as two standard deviations based on at least three different experiments.

b H-Bond acceptor parameter (*β*) of the corresponding 4-X-pyridine from ref. 9.

c The measured association constant involves breaking an intramolecular H-bond, so the apparent *α* value cannot be compared.

### H-bond parameters

The UV-vis titration data for each of compounds 2–7 with tri-*n*-butyl phosphine oxide fit well to a 1:2 binding isotherm, and in all cases, the association constant for the second binding interaction was less than 10 M^−1^ (see ESI†). The association constants for formation of the 1:1 complex (*K*_1_) are listed in Table 2. Comparison with the H-bond acceptor parameters of the corresponding 4-X-pyridines, *β*(pyridine), shows that the association constant for formation of the intermolecular H-bond decreases as the strength of the intramolecular H-bond increases. This negative allosteric cooperativity can be quantified by converting the association constants in Table 2 to H-bond donor parameters *α*(aniline) using eqn (1).^4^

1 Δ*G*/kJ mol^−1^ = −*RT* ln *K*_1_ = −(*α* − *α*_S_)(*β* − *β*_S_) + 6

where *β* is the H-bond acceptor parameter for tri-*n*-butyl phosphine oxide (10.7),^35^*α* is the H-bond donor parameter of the aniline group, and *α*_S_ and *β*_S_ are the H-bond parameters of the solvent (1.2 and 0.6 respectively for *n*-octane).^36^

**Fig. 9:**
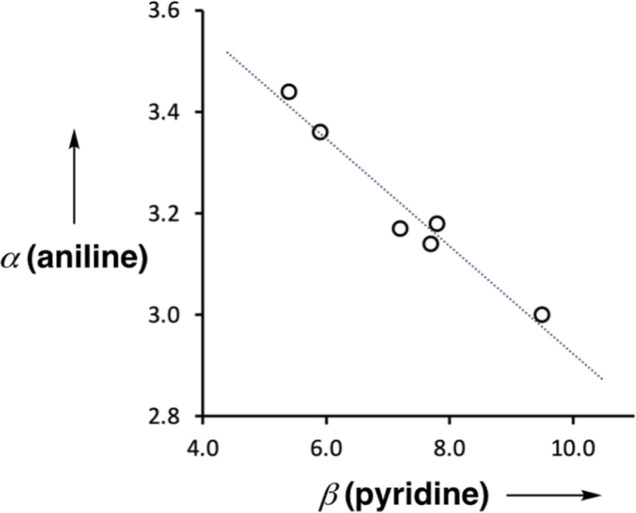
Relationship between the H-bond donor parameters of compounds 2–7, *α*(aniline), and the H-bond acceptor properties of the corresponding 4-X-pyridines, *β*(pyridine). The best fit line is *y* = −0.10*x* + 3.97 (*R*^2^ = 0.96).

Fig. 9 shows the relationship between the value of *α*(aniline) measured for compounds 2–7 and the value of *β*(pyridine) for the corresponding 4-X-pyridines. There is a linear relationship, and the slope of −0.10 corresponds to the cooperativity parameter, *κ*, for the aniline functional group.^6^ This parameter can be compared with the values that we have measured previously for phenols (+0.33) and amides (+0.20),^6,8,9^ which show positive allosteric cooperativity. The magnitude of the cooperative effect is lower in aniline, and it is negative rather than positive.

The positive cooperativity observed in amides is related to polarisation of the functional group in the presence of a H-bond, such that the oxygen becomes more negative and the NH hydrogen becomes more positive.^9^ It is possible that polarisation of the *p*-nitroaniline group in compounds 2–7 would result in a similar effect, *i.e.* a more negative nitro group and a more positive amino group. However, such polarisation would lead to positive cooperativity, so the cooperative effects measured in this system must be related to the local properties of the primary amine group. There are two possible explanations. Formation of the intramolecular H-bond could polarise the NH bond, such that the hydrogen becomes more positive and the nitrogen more negative, which would reduce the polarity of the second NH donor. Alternatively, there could be secondary electrostatic interactions between the two H-bond acceptors, leading to repulsion when they are held in close proximity, bound to the same amine group. The observed negative cooperativity may be due to a combination of these two effects.

**Fig. 10:**
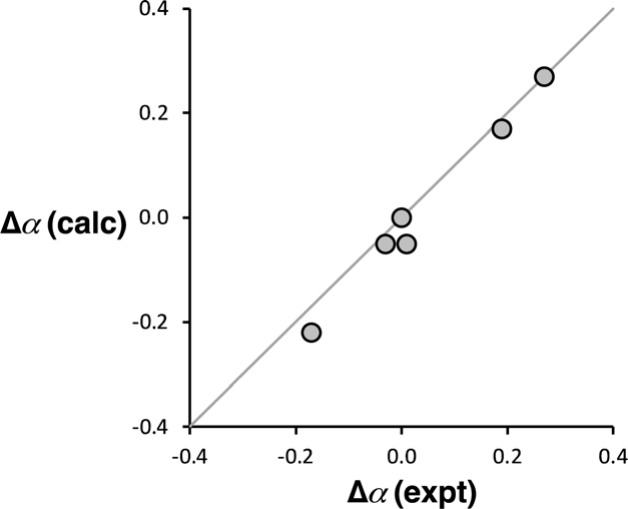
Comparison of the calculated and experimental values of substituent effects on the H-bond donor parameters of compounds 2–7 (Δ*α* = *α*_X_ − *α*_H_). The line corresponds to *y* = *x*.

Computational chemistry was used to investigate whether the magnitude of the cooperative effects measured for compounds 2–7 could be rationalised using electrostatics. The molecular electrostatic potential calculated on the 0.0104 e Bohr^−3^ electron density isosurface using density functional theory (DFT) was used to calculate values of *α* for compounds 2–7 (see ESI† for full details).^37^ The results are compared with the *α* values in Fig. 10. The calculated molecular electrostatic potential surface of an aniline is rather sensitive to the degree of pyramidalization obtained from a structure optimisation, and the calculated values of *α* were consistently underpredicted by about 0.2. Nevertheless, the effect of the X substituents on the values of *α* (Δ*α* = *α*_X_ − *α*_H_) are extremely well reproduced in the calculations (Fig. 10). The DFT calculations capture both the change in polarity of the NH group and through space electrostatic interactions with the pyridine H-bond acceptor, so although there two effects cannot be easily separated, it is clear that the observed negative cooperativity can be explained in a quantitative manner based on electrostatic effects.

## Conclusions

Compounds with an intramolecular H-bond between an aniline NH_2_ group and a pyridine nitrogen have been used to investigate the effect of cooperativity on the H-bond donor properties of primary amines. X-ray crystallography, downfield changes in ^1^H NMR chemical shift, and ^1^H–^15^N HMBC experiments in *n*-octane were used to confirm the presence of an intramolecular H-bond with one of the two aniline NH protons. ^1^H NMR titration experiments showed that tri-*n*-butyl phosphine oxide binds to the other aniline NH proton to form a 1:1 complex, in which the amino groups forms an intramolecular H-bond with the pyridine H-bond acceptor and an intermolecular H-bond with the phosphine oxide H-bond acceptor. UV-vis absorption titrations were used to measure the association constants for formation of 1:1 complexes with a series of anilines in which the strength of the intramolecular H-bond was varied using polarising substituents on the pyridine ring. Electron-donating substituents, which increase the strength of the intramolecular H-bond, were found to decrease the strength of the intermolecular H-bond between the aniline and the phosphine oxide.

The results were used to determine the H-bond donor parameters for the anilines, *α*, and there is a linear relationship between the values of *α* and the H-bond acceptor parameter of the pyridine group involved in the intramolecular H-bond, *β*. The slope of this relationship was used to determine the cooperativity parameter (*κ* = −0.10), which quantifies the negative allosteric cooperativity between the two H-bonding interactions. The cooperativity parameter measured here for anilines is of the opposite sign and lower in magnitude than the values previously reported for amides (*κ* = 0.20)^9^ and phenols (*κ* = 0.33).^6^ Molecular electrostatic potential surfaces calculated using DFT quantitatively reproduce the negative cooperativity measured in the anilines, which suggests that effects are electrostatic in origin, either due to polarisation of the NH bonds or due to secondary electrostatic interactions between the two H-bond acceptors.

## Data availability

All supporting data is provided in the ESI.†

## Author contributions

The manuscript was written through contributions of all authors.

## Conflicts of interest

There are no conflicts to declare.

## Supplementary Material

SC-015-D4SC03719G-s001

SC-015-D4SC03719G-s002
