# Supplementary material for: Negative cooperativity in the formation of H-bond networks involving primary anilines
Source: Chem Sci. 2024 Jul 3;15(30):12036–41. doi: 10.1039/d4sc03719g (PMC11290332; doi:10.1039/d4sc03719g)
Supplement: SC-015-D4SC03719G-s001 [file SC-015-D4SC03719G-s001.pdf]

Electronic Supplementary Material (ESI) for Chemical Science.
This journal is © The Royal Society of Chemistry 2024

Negative Cooperativity in the Formation of H-Bond Networks involving Primary Anilines

Fergal E. Hanna,[a] Alexander J. Root,[a] Markus Schade,[b] and Christopher A. Hunter*,[a]

[a] *Yusuf Hamied Department of Chemistry, University of Cambridge, Lensfield Road, Cambridge CB2 1EW, UK.*

[b] *Chemistry, Oncology R&D, AstraZeneca, 1 Francis Crick Avenue, Cambridge CB2 0AA, UK.*

E-mail: herchelsmith.orgchem@ch.cam.ac.uk

Supplementary Information

Table of Contents

Materials and methods 2
Synthesis and characterisation 3
X-ray crystallography 12
NMR spectra of anilines in *n*-octane 14
UV-vis absorption and ^{1}H NMR titration data 16
Results from fitting of titration data 41
DFT calculations 42
References 43

Materials and methods

All reagents were purchased from commercial sources (Sigma Aldrich UK, Acros, Tokyo Chemical Industry, Alfa Aesar, Manchester Organics and FluoroChem) and were used as received without any further purification unless stated. Dry solvents were obtained by means of a Grubbs solvent purification system.

Flash chromatography was done with an automated system (Combiflash Companion) using pre-packed cartridges of silica (50 µm PuriFlash® column) or basic alumina (45 µm PuriFlash® column)

The LC-MS analysis of samples was performed using Waters Acquity H-class UPLC coupled with a single quadrupole Waters SQD2. ACQUITY UPLC CSH C18 Column, 130 Å, 1.7 µm, 2.1 mm X 50 mm was used as the UPLC column for all samples. The conditions of the UPLC method are as follows: Solvent A: Water +0.1% Formic acid; Solvent B: Acetonitrile +0.1% Formic acid; Gradient of 0-2 minutes 5% - 100%B + 1 minute 100% B with re-equilibration time of 2 minutes. Flow rate: 0.6 ml/min; column temperature of 40°C; injection volume of 2 µL. The signal was monitored with MS-ES+, MS-ES-, and UV-vis absorption at 254 nm or at 290 nm.

^{1}H-NMR and ^{13}C-NMR were recorded on either a 400 MHz or 500 MHz Bruker spectrometer. ^{15}N NMR experiments were carried out using a 500 MHz NMR spectrometer equipped with a TCI cryoprobe head. The reference values used for the chemical shifts of the various spectra are reported in the literature.[1] The splitting pattern is indicated with the following abbreviations: s for singlet, d for doublet, t for triplet, q for quartet, p for pentet and m for multiplet.

FT-IR spectra were collected with a Bruker ALPHA FT-IR Spectrometer.

UV-vis spectra were recorded with an Agilent UV-vis Cary 60 spectrophotometer.

Melting points were measured in a Mettler Toledo MP50 Melting Point System.

Synthesis

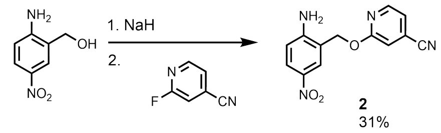

Scheme 1 – Synthesis of **2**.

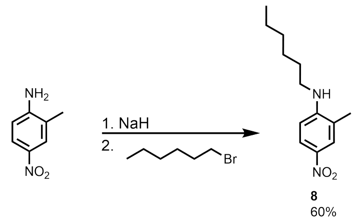

Scheme 2- Synthesis of **8**.

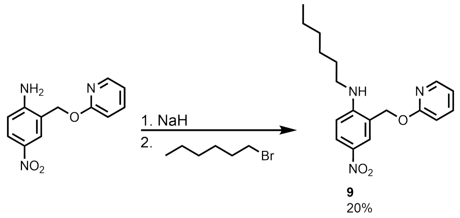

Scheme 3 -Synthesis of **9.** Synthesis of starting material reported previously by our group.[2]

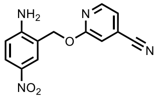

2-((2-amino-5-nitrobenzyl)oxy)isonicotinonitrile, **2**. Sodium hydride (222 mg, 60% in mineral oil, 5.55 mmol) and 2-amino-5-nitrobenzyl alcohol (416 mg, 2.47 mmol) were dissolved in dry DMF (2.5 mL) under N_2. 2-fluoro-4-cyano pyridine (290 mg, 2.38 mmol) was dissolved in dry DMF (0.5 mL) under N_2 and then added to the reaction mixture, which was subsequently stirred for one week, before the reaction was quenched with methanol (5 mL). The organic components were extracted with ethyl acetate (50 mL) and filtered. A portion of this mixture (25mL) was washed with saturated $NaHCO_3$ (25 mL), brine (25 mL), water (2 × 25 mL) and 5% LiCl (25 mL). The organic extracts were dried with $MgSO_4$, and the solvent removed under reduced pressure. The crude product was purified by flash chromatography (PE/EtOAc on SiO_2) to yield the desired product **2** as a yellow solid (101 mg, 0.37 mmol, 31 %).

ν_{max} (film) cm^{-1} : 3481 (N-H), 3461 (N-H), 1634 (C=C), 1603 (C=C), 1588 (C=C), 1549 (C=C), 1508 (C=C), 1492 (C=C), 1411, 1316, 1284, 1239, 1149, 1101, 1015, 984, 824, 750, 643, 491, 431.

^1H NMR (400 MHz, DMSO-d_6) δ_H ppm: 8.44 (d, J = 5.3 Hz, 1H), 8.12 (d, J = 2.7 Hz, 1H), 7.96 (dd, J = 9.0, 2.8 Hz, 1H), 7.49 (d, J = 6.3 Hz, 1H), 7.44 (d, J = 5.2 Hz, 1H), 6.79 – 6.71 (m, 3H), 5.30 (s, 2H).

$^{13}C\{^1H\}$ NMR (101MHz, DMSO-d_6) δ_C ppm: 163.1, 153.9, 148.5, 135.5, 126.8, 125.9, 121.8, 118.4, 118.0, 116.6, 114.3, 113.8, 64.6.

HRMS: calc for $C_{13}H_{11}N_4O_3^+$ $[M+H]^+$: 271.0826, found: 271.0836.

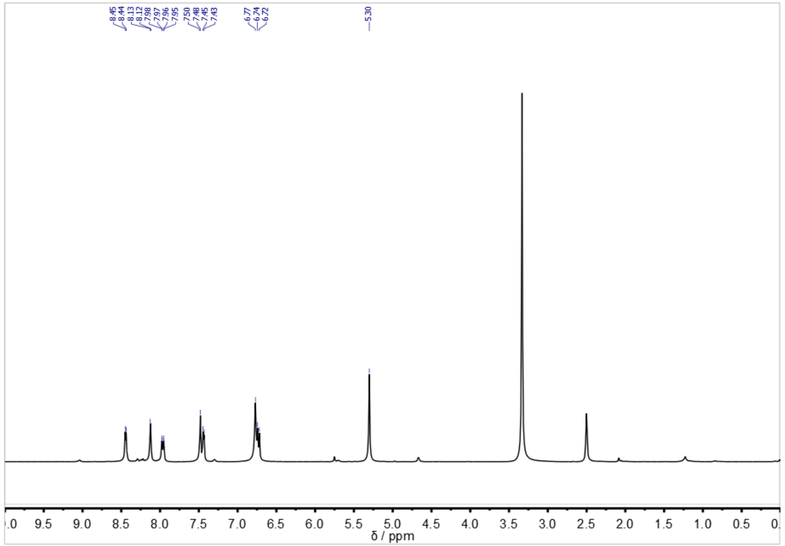

Figure S.1 – 400 MHz ^1H NMR spectrum of **2** in DMSO-d_6 (δ 0 – 10 ppm).

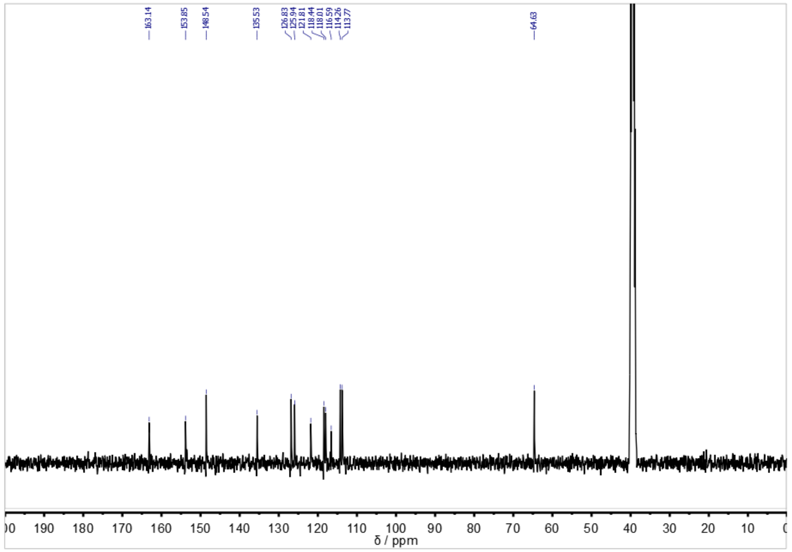

Figure S.2 – 101 MHz ^{13}C NMR spectrum of **2** in DMSO-d_6 (δ 0 – 200 ppm).

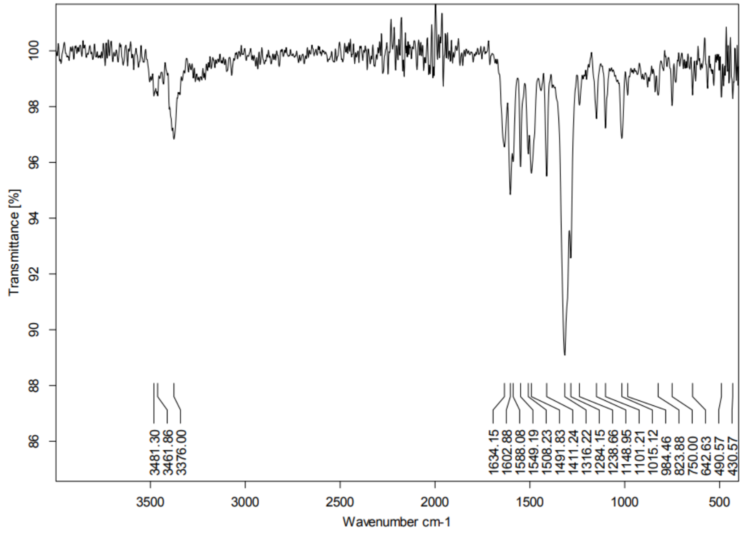

Figure S.3 – FT-IR spectrum of **2**.

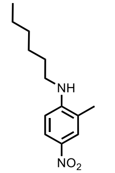

N-hexyl-2-methyl-4-nitroaniline, **8**. Sodium hydride (60% in mineral oil, 90.7 mg, 2.268 mmol) was washed with petroleum ether (3 x 5 mL) and then suspended in dry DMF (2 mL) under N_2. 2-methyl-4-nitroaniline (308 mg, 2.02 mmol) was dissolved in dry DMF (2 mL) under N_2, added the sodium hydride suspension and stirred for 1 hour. 1-bromohexane (0.30 mL, 2.1 mmol) was added and the reaction stirred for 24 hours. The reaction mixture was quenched with EtOH (10 mL) and diluted with EtOAc (50 mL). The mixture was then washed with saturated NH_4Cl solution (25 mL), water (25 mL), 5% LiCl solution (25 mL), and brine (25 mL). The organic layer was dried with $MgSO_4$ and the solvent removed under reduced pressure. The crude residue was purified using flash chromatography to yield the desired product as a yellow solid (288 mg, 1.22 mmol, 60%).

ν_{max} (film) cm^{-1} : 3410 (N-H), 2951 (C-H), 2932 (C-H), 2862 (C-H), 1601 (C=C), 1589 (C=C), 1530 (C=C), 1494 (C=C), 1474 (C=C), 1464 (C=C), 1317, 1306, 1278, 1185, 1102.

^{1}H NMR (400 MHz, $CDCl_3$) δ_H ppm: 8.06 (dd, J = 9.0, 2.6 Hz, 1H), 7.97 (d, J = 2.6 Hz, 1H), 6.53 (d, J = 9.0 Hz, 1H), 4.24 (s, 1H), 3.25 (t, J = 7.2 Hz, 2H), 2.17 (s, 3H), 1.69 (p, J = 7.2 Hz, 2H), 1.48 – 1.39 (m, 2H), 1.34 (dq, J = 7.2, 3.6 Hz, 4H), 0.98 – 0.85 (m, 3H).

^{13}C{^{1}H} NMR (101MHz, $CDCl_3$) δ_C ppm: 151.8, 137.4, 126.2, 124.9, 120.8, 107.8, 43.7, 31.7, 29.3, 26.87, 22.7, 17.4, 14.1.

HRMS: calc for $C_{13}H_{21}N_2O_2^+$ [M+H]$^+$: 237.1603, found: 237.1607.

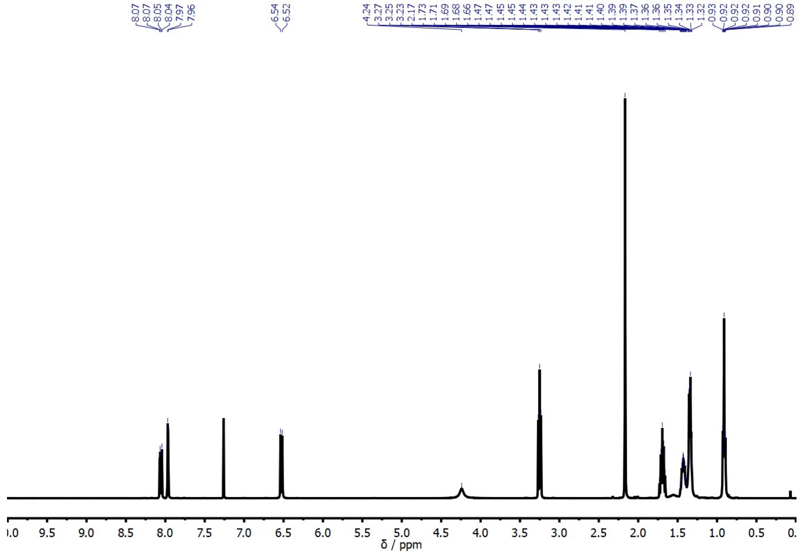

Figure S.4 – 400 MHz ^{1}H NMR spectrum of **8** in $CDCl_3$ (δ 0 – 10 ppm).

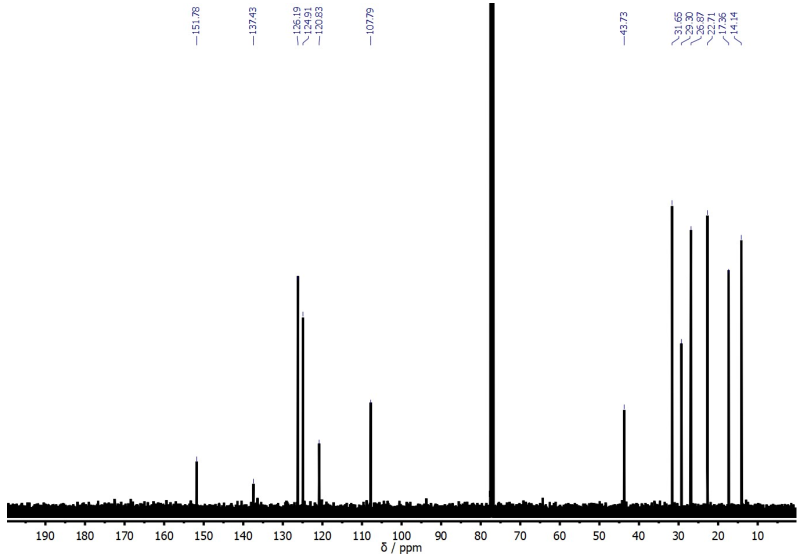

Figure S.5 – 101 MHz ^{13}C NMR spectrum of **8** in $CDCl_3$ (δ 0 – 200 ppm).

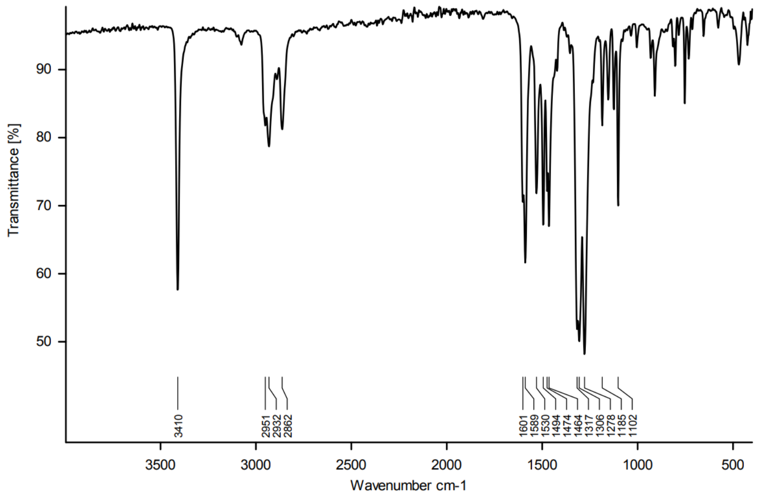

Figure S.6 – FT-IR spectrum of **8**.

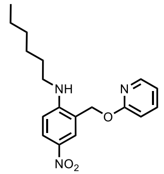

N-hexyl-4-nitro-2-((pyridin-2-yloxy)methyl)aniline, **9**. 4-nitro-2-((pyridin-2-yloxy)methyl)aniline (77.1 g, 0.314 mmol), **4**, and sodium hydride (60% in mineral oil, 15.7 mg, 0.393 mmol) were dissolved in dry DMF (1 mL) under a nitrogen atmosphere and stirred for 15 minutes. 1-bromohexane (50 µL, 0.36 mmol) was added and the reaction stirred overnight. The reaction was quenched with EtOH (5 mL) and diluted in EtOAc (50 mL). The mixture was washed with saturated NH_4Cl (10 mL), water (2 x 10 mL), 5% LiCl solution (10 mL) and brine (10 mL). The organic layer was dried with $MgSO_4$ and the solvent removed under reduced pressure. The crude residue was purified by flash chromatography (PE/EtOAc on SiO_2) to yield the desired product as a yellow solid (21.2 mg, 0.0643 mmol, 20%).

ν_{max} (film) cm^{-1}: 3421 (N-H), 3285. 2954 (C-H), 2928 (C-H), 2857 (C-H), 1593 (C=C), 1571 (C=C), 1497 (C=C), 1472 (C=C), 1430, 1323, 1284, 1232, 1098, 989, 976, 779, 752.

^{1}H NMR (400 MHz, $CDCl_3$) δ_H ppm: 8.22 (d, J = 2.7 Hz, 1H), 8.17 (dd, J = 5.0, 1.4 Hz, 1H), 8.14 (dd, J = 9.1, 2.7 Hz, 1H), 7.61 (ddd, J = 8.8, 7.1, 2.0 Hz, 1H), 6.94 (ddd, J = 7.2, 5.0, 0.9 Hz, 1H), 6.77 (dd, J = 8.3, 1.0 Hz, 1H), 6.56 (d, J = 9.2 Hz, 1H), 6.23 (t, J = 5.1 Hz, 1H), 5.40 (s, 2H), 3.22 (td, J = 7.0, 5.0 Hz, 2H), 1.65 (p, J = 7.1 Hz, 2H), 1.37 (pd, J = 6.3, 3.1 Hz, 2H), 1.31 (dt, J = 7.8, 3.9 Hz, 4H), 0.89 (td, J = 5.9, 4.8, 3.2 Hz, 3H).

^{13}C{^{1}H} NMR (101MHz, $CDCl_3$) δ_C ppm: 163.4, 152.9, 146.6, 146.6, 139.4, 136.9, 128.1, 127.1, 120.0, 117.8, 111.7, 109.0, 65.6, 43.6, 31.7, 29.0, 26.9, 22.7, 14.1.

HRMS: calc for $C_{18}H_{24}N_3O_3^+$ [M+H]$^+$: 330.1818, found: 330.1802.

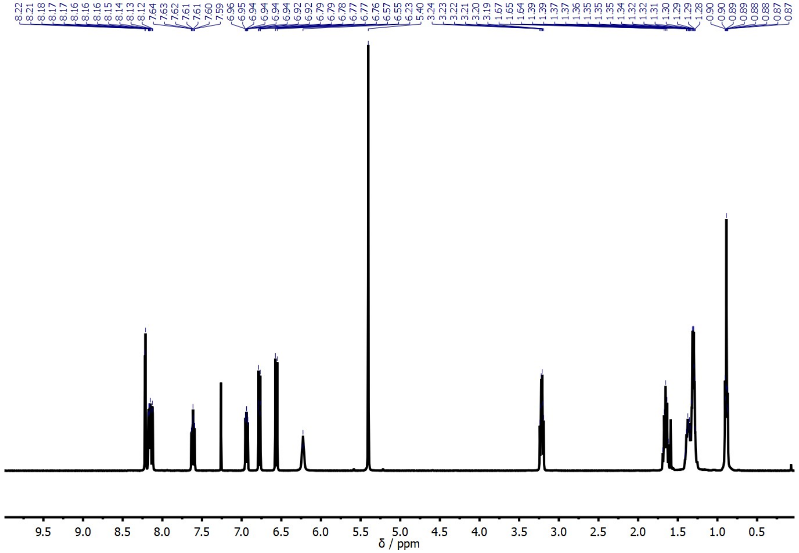

Figure S.7 – 400 MHz ^{1}H NMR spectrum of **9** in $CDCl_3$ (δ 0 – 10 ppm).

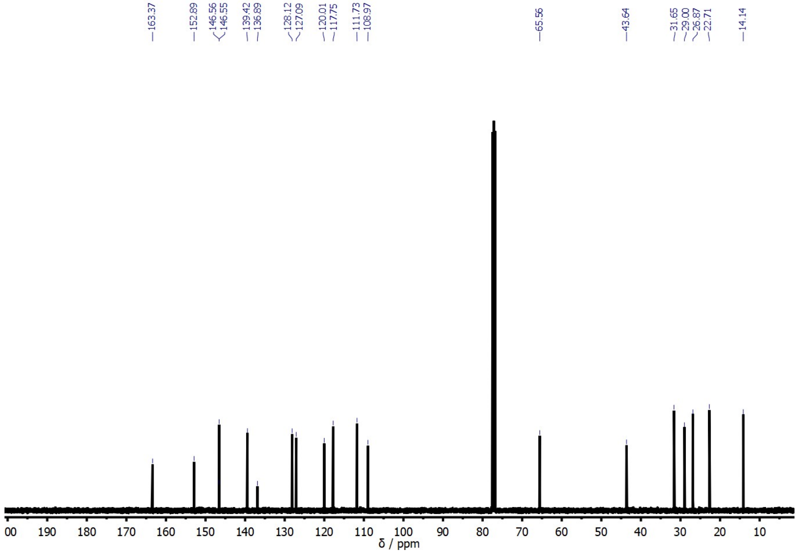

Figure S.8 – 101 MHz ^{13}C NMR spectrum of **9** in $CDCl_3$ (δ 0 – 200 ppm).

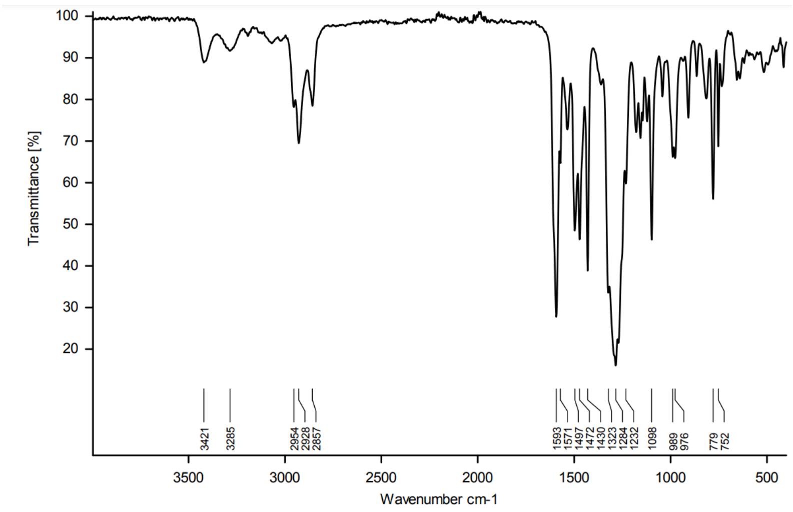

Figure S.9 – FT-IR spectrum of **9**.

X-ray crystallography

X-ray data were collected on a Bruker D8-QUEST diffractometer, equipped with an Incoatec IµS Cu microsource (λ = 1.5418 Å) and a PHOTON-III detector operating in shutterless mode. The crystal temperature was held at 180(2) K using an Oxford Cryosystems open-flow N2 Cryostream. The control and processing software was Bruker APEX4 (ver. 2021.4-0). Structures were solved using SHELXT[3] and refined using SHELXL.[4]

Structure **9** (displacement ellipsoids at 50% probability):

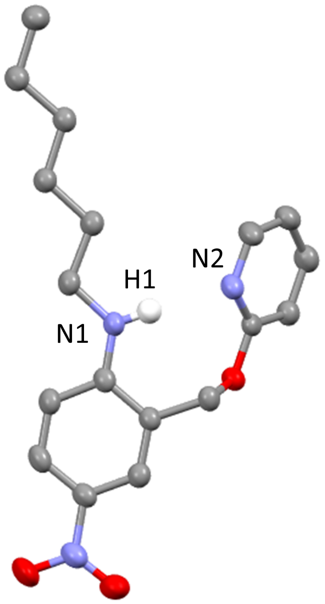

Figure S.10 – Crystal structure of compound **9** determined by X-ray diffraction. Ellipsoids at 50% probability.

D	H	A	D-H (Å)	H···A (Å)	D···A (Å)	D-H···A (°)
N1	H1	N2	0.900	2.084	2.965	165.86

Table S.1 – Measurement of distances and angles involved in the intramolecular H-bond in compound **9**.

Cambridge Identification code	CH_B1_0075
Chemical formula	$C_{18}H_{23}N_3O_3$
Formula weight	329.39
Temperature / K	180(2)
Crystal system	Monoclinic
Space group	$P2_1/c$
a / Å	a = 17.6378(9) Å

b / Å	b = 4.1739(3) Å
c / Å	c = 24.0146(11) Å
α / °	90
β / °	106.817(3)
γ / °	90
Volume / Å^3	1692.31(17)
Z	4
Density (calculated) / g cm^{-3}	1.293
Absorption coefficient / mm^{-1}	0.724
F(000)	704
Crystal size / mm^3	0.280 x 0.080 x 0.020
2-theta range / °	2.617 to 66.608
Index ranges	-20<=h<=20, -4<=k<=4, -28<=l<=28
No. of reflections collected	22417
No. of independent reflections	2963
R(int)	0.1147
Completeness to theta = 66.608°	100.0 %
Absorption correction	Semi-empirical from equivalents
Max. and min. transmission	0.7528 and 0.5982
Refinement method	Full-matrix least-squares on F^2
No. of parameters / restraints	223 / 0
Goodness-of-fit on F^2	1.029
Final R indices [I>2sigma(I)]	R1 = 0.0520, wR2 = 0.1164
R indices (all data)	R1 = 0.0823, wR2 = 0.1349
Extinction coefficient	0.0031(5)
Largest diff. peak and hole	0.202 and -0.238 e.Å^{-3}

Table S.2 – Summary of the crystal and refinement details.

NMR spectra anilines in *n*-octane

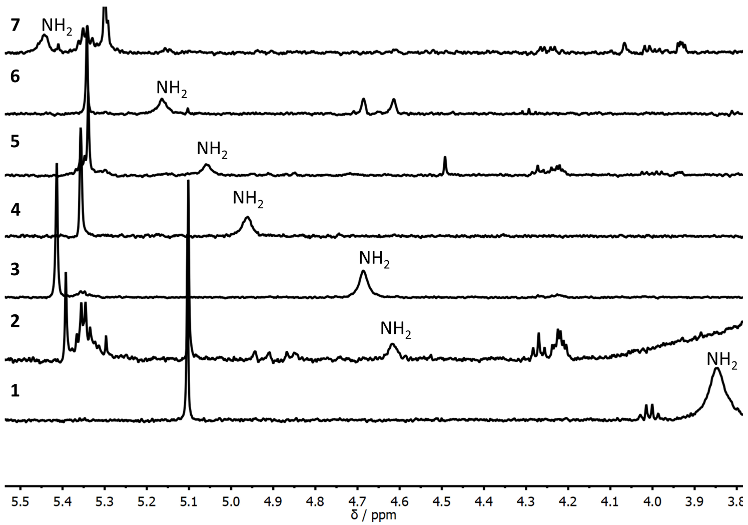

Figure S.11 – Partial 400 MHz ^{1}H NMR spectra of compounds **1-7** in *n*-octane at 298 K (0.0050 – 0.35 mM).

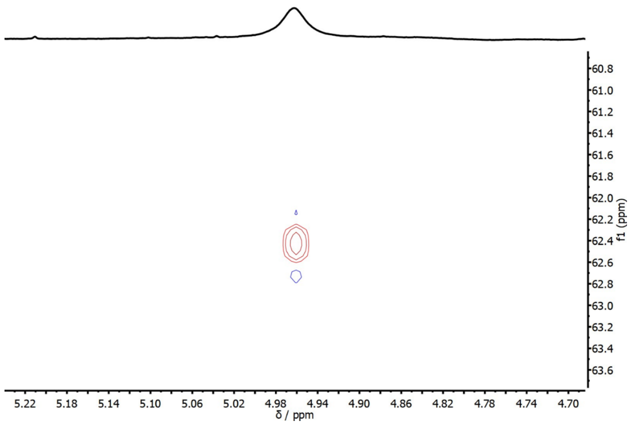

Figure S.12 – Partial 500 MHz ^{15}N-^{1}H HSQC spectrum of compound **4** (0.31 mM in *n*-octane) recorded with the 1-bond N-H coupling constant set to 90 Hz. The cross-peak is between the signal due to the aniline protons and the signal due to the aniline nitrogen.

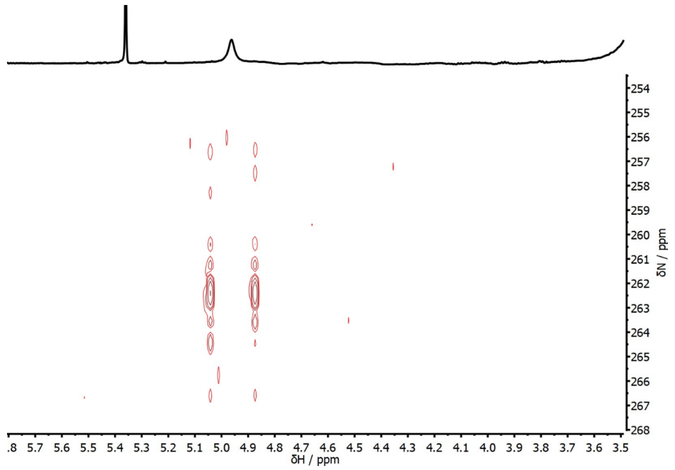

Figure S.13 – Partial 500 MHz ^{15}N-^{1}H HMBC spectrum of compound **4** (0.31 mM in *n*-octane) recorded with the long-range N-H coupling constant set to 8 Hz. The cross-peak is between the signal due to the aniline protons and the signal due to the pyridine nitrogen.

UV-vis Absorption and NMR Titrations

UV-vis titrations were carried out on an Agilent Cary 60 UV-Vis spectrophotometer, using standard titration protocols. A sample of the host (**1-9**) was prepared at a known concentration (typically between 0.02-0.06 mM) in *n*-octane. The UV-vis spectrum of the free host (2 mL) was recorded. The guest was dissolved (Bu_3PO) in 2 mL of the host solution at a known concentration. Aliquots of the guest solution were successively added to the cuvette, and the UV-vis absorption spectrum was recorded after each addition. The UV-vis absorption spectra were analysed using a purpose-built Python script to fit the changes in the absorption at fixed wavelengths to either a 1:1 or a 1:2 binding isotherm by optimizing the association constant and absorption of the free and bound host.

NMR titrations were carried out on a Bruker 500 MHz spectrometer, using standard titration protocols. A sample of the host was prepared at a concentration of 0.3-0.8 mM in *n*-octane. The NMR spectrum of the host solution (0.6 mL) was recorded. The guest (Bu_3PO) was dissolved in 2 mL of the host solution at a known concentration. Aliquots of the guest solution were successively added to the NMR sample tube containing the host solution, and the ^{1}H NMR spectrum was recorded after each addition. For the dilution experiment, 0.6 mL of the host in *n*-octane was placed in an NMR tube and aliquots of a solution pure *n*-octane was added with the spectrum being recorded after every addition. The NMR spectra were analysed using a purpose-built Python script to fit the changes in the chemical shifts for different protons to either a 1:1 or a 1:2 binding isotherm by optimizing the association constant and shifts of the free and bound host.

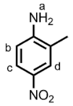

Figure S.14 – Structure of the host **1** with protons labelled for NMR assignment.

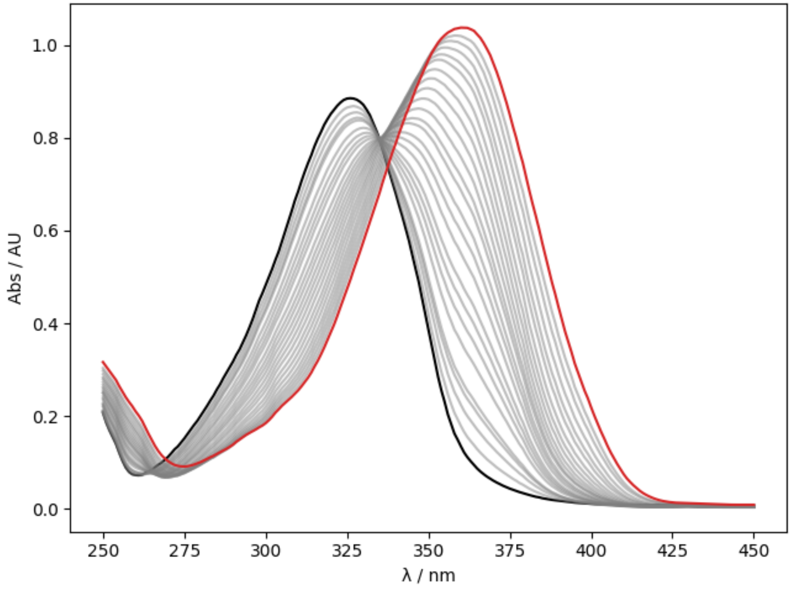

Figure S.15 – UV-vis absorption spectra for the titration of Bu_3PO into **1** (0.0503 mM in *n*-octane, at 298 K). The UV-vis spectrum of the host **1** and the final point of the titration are reported in black and in red, respectively.

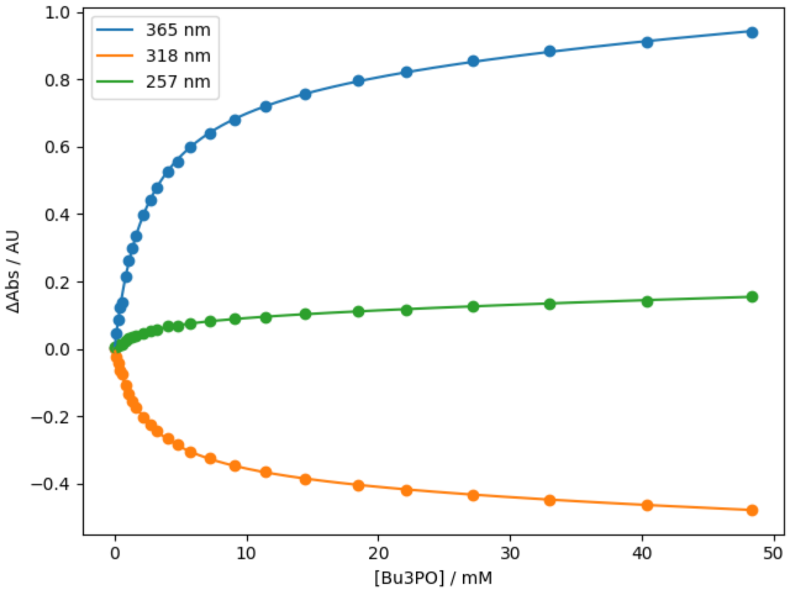

Figure S.16 – The fit of the absorbance at selected wavelengths to a 1:2 binding isotherm for the titration of Bu_3PO into **1** (0.0503 mM in *n*-octane, at 298 K).

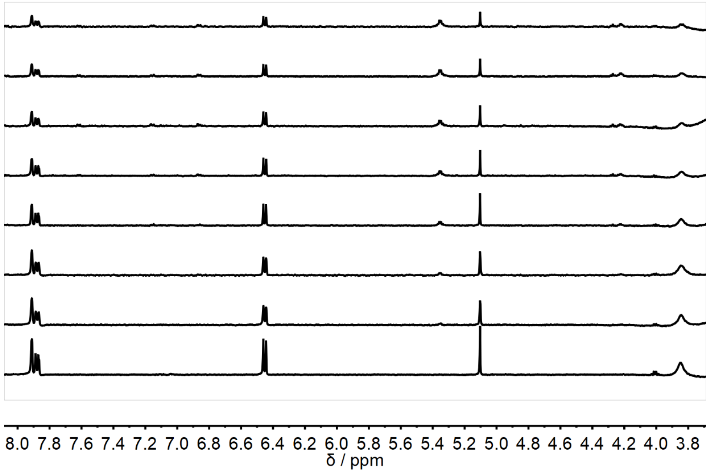

Figure S.17 – Stack plot for the 500 MHz ^{1}H NMR dilution of **1** in *n*-octane at 298 K ranging from 0.414 mM (bottom) to 0.124 mM (top).

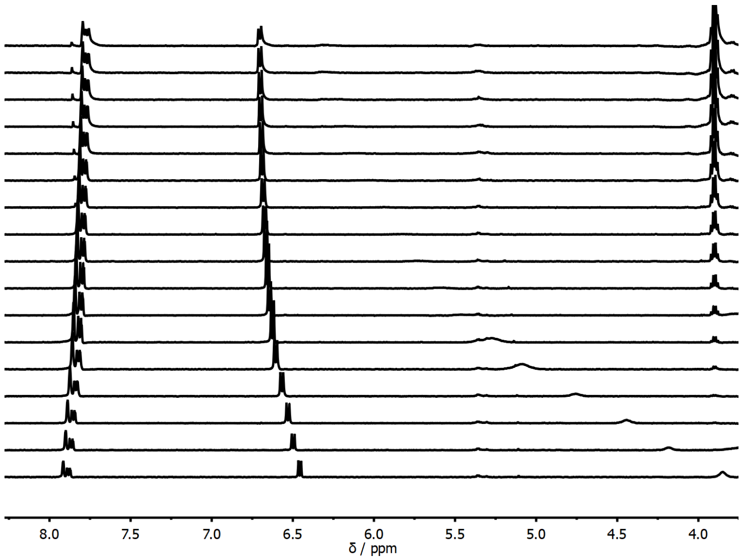

Figure S.18 – Stack plot for the 500 MHz ^{1}H NMR titration of Bu_3PO into **1** (0.414 mM) in *n*-octane at 298 K.

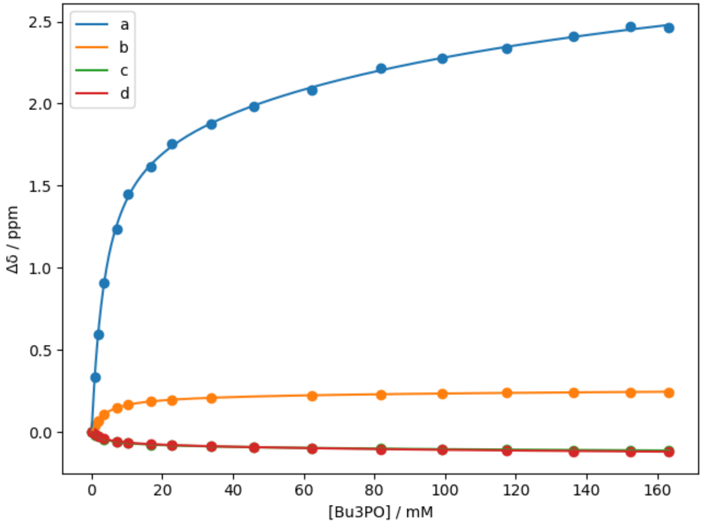

Figure S.19 – Fit of the chemical shift changes to a 1:2 binding isotherm for the NMR titration of Bu_3PO into **1** (0.310 mM) in *n*-octane at 298 K.

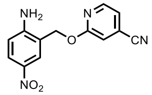

Figure S.20– Structure of the host **2**.

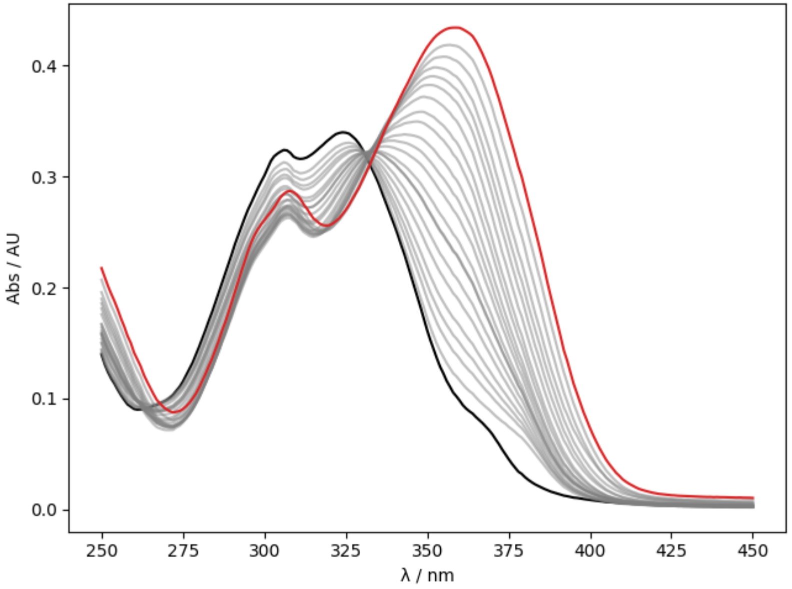

Figure S.21 – UV-vis absorption spectra for the titration of Bu_3PO into **2** (0.0562 mM in *n*-octane, at 298 K). The UV-vis spectrum of the host **2** and the final point of the titration are reported in black and in red, respectively.

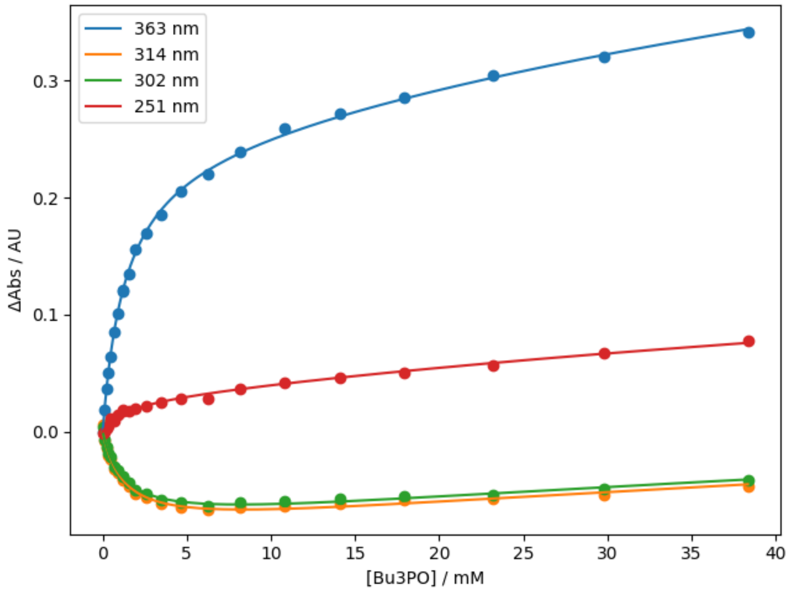

Figure S.22 – The fit of the absorbance at selected wavelengths to a 1:2 binding isotherm for the titration of Bu_3PO into **2** (0.0562 mM in *n*-octane, at 298 K).

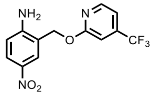

Figure S.23 – Structure of the host **3**.

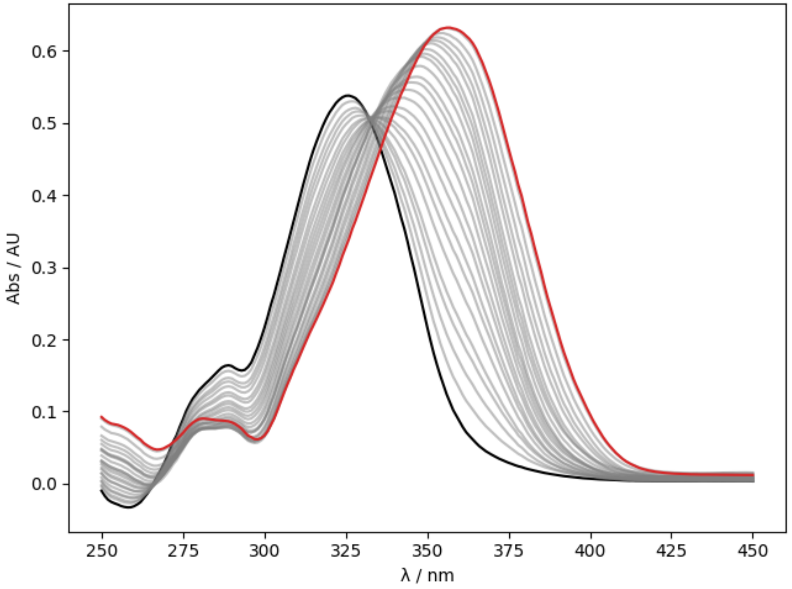

Figure S.24 – UV-vis absorption spectra for the titration of Bu_3PO into **3** (0.0479 mM in *n*-octane, at 298K). The UV-vis spectrum of the host **3** and the final point of the titration are reported in black and in red, respectively.

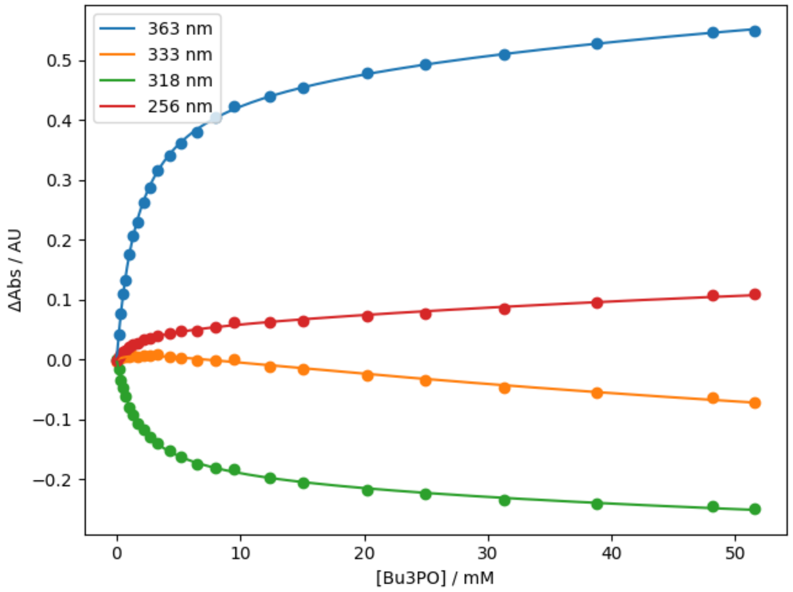

Figure S.25 – The fit of the absorbance at selected wavelengths to a 1:2 binding isotherm for the titration of Bu_3PO into **3** (0.0254 mM in *n*-octane, at 298 K).

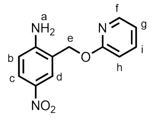

Figure S.26 – Structure of the host **4** with protons labelled for NMR assignment.

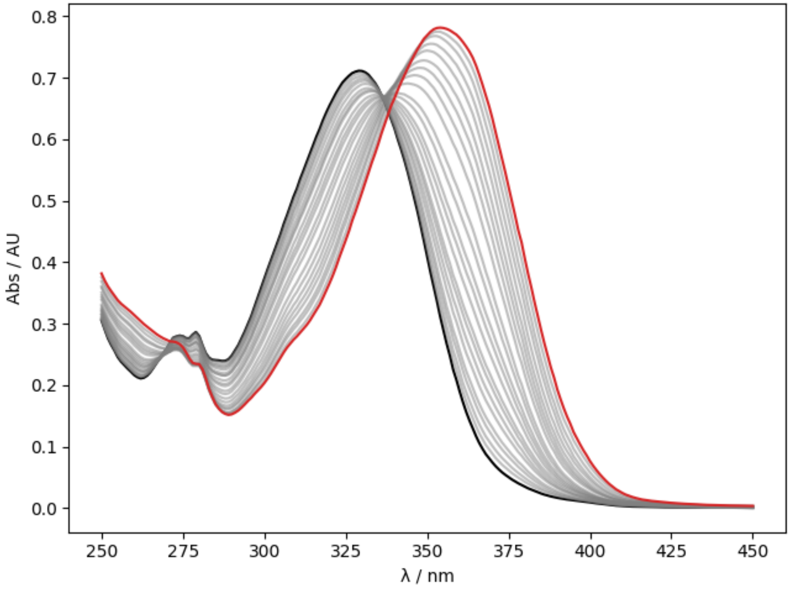

Figure S.27 – UV-vis absorption spectra for the titration of Bu_3PO into **4** (0.0496 mM in *n*-octane, at 298K). The UV-vis spectrum of the host **4** and the final point of the titration are reported in black and in red, respectively.

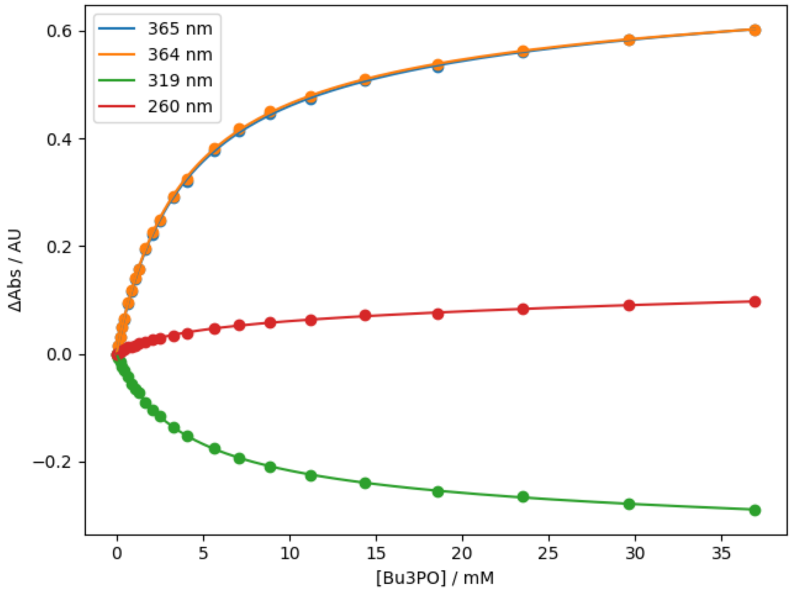

Figure S.28 – The fit of the absorbance at selected wavelengths to a 1:2 binding with guest absorption isotherm for the titration of Bu_3PO into **4** (0.0496 mM in *n*-octane, at 298 K).

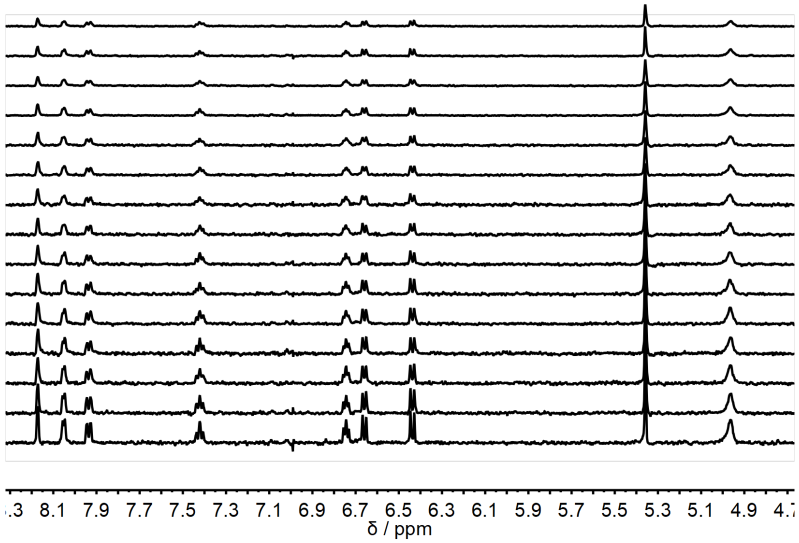

Figure S.29 – Stack plot for the 500 MHz ^{1}H NMR dilution of **4** in *n*-octane at 298 K ranging from 0.310 mM (bottom) to 0.0715 mM (top).

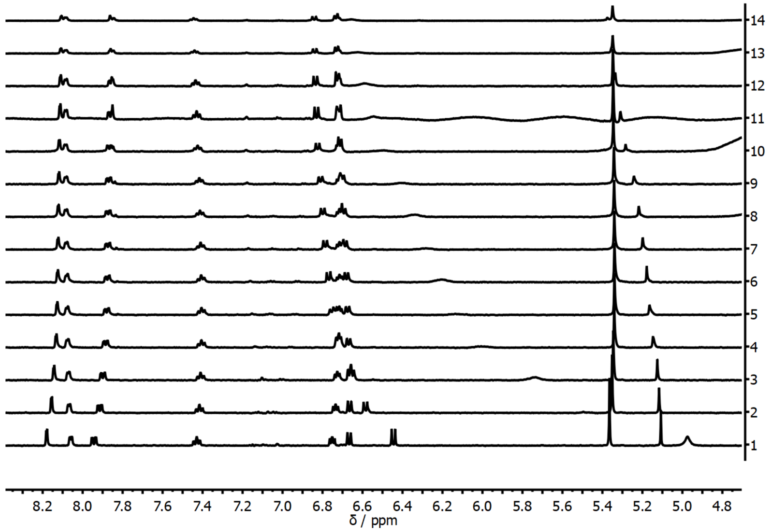

Figure S.30 – Stack plot for the 500 MHz ^{1}H NMR titration of Bu_3PO into **4** (0.310 mM) in *n*-octane at 298 K.

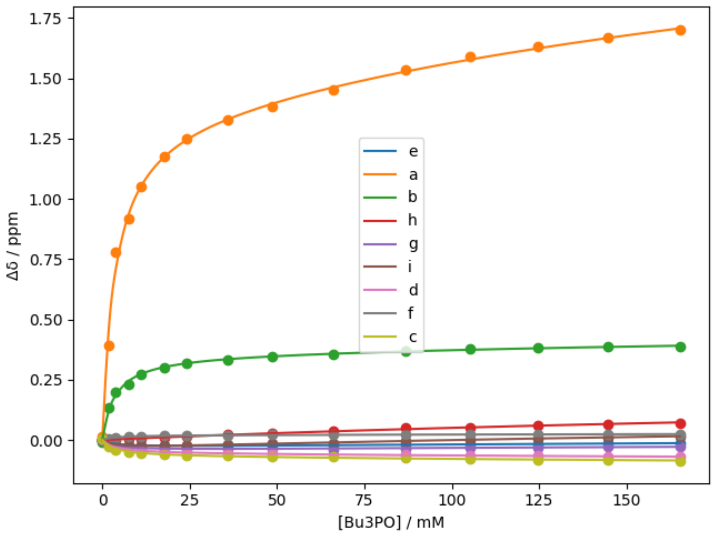

Figure S.31 – Fit of the chemical shift changes to a 1:2 binding isotherm for the NMR titration of Bu_3PO into **4** (0.310 mM) in *n*-octane at 298 K.

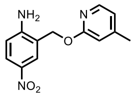

Figure S.32 – Structure of the host **5**.

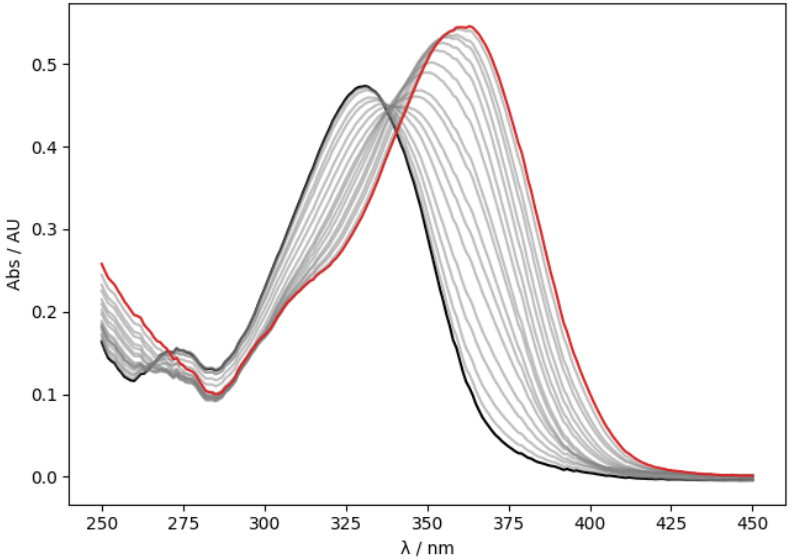

Figure S.33 – UV-vis absorption spectra for the titration of Bu_3PO into **5** (0.0459 mM in *n*-octane, at 298 K). The UV-vis spectrum of the host **5** and the final point of the titration are reported in black and in red, respectively.

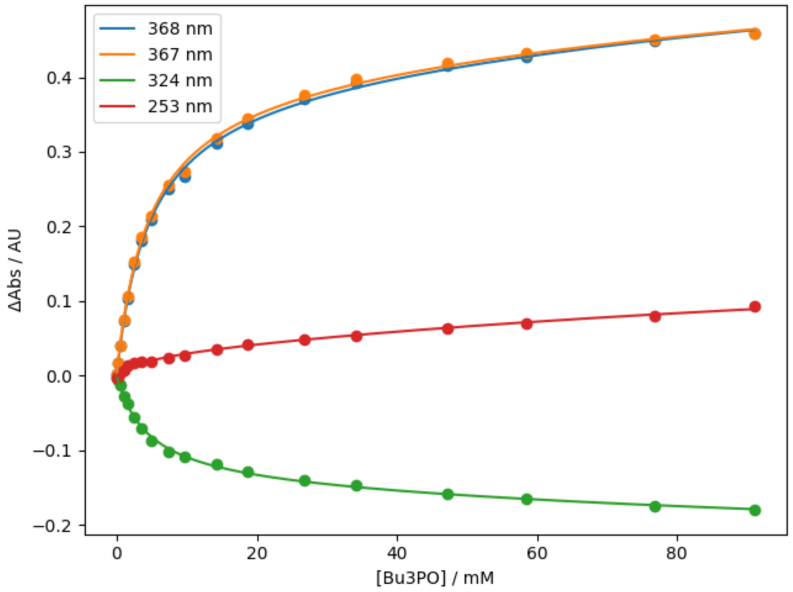

Figure S.34 - The fit of the absorbance at selected wavelengths to a 1:2 binding with guest absorption isotherm for the titration of Bu_3PO into **5** (0.0459 mM in *n*-octane, at 298 K).

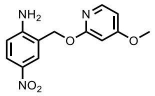

Figure S.35 – Structure of the host **6**.

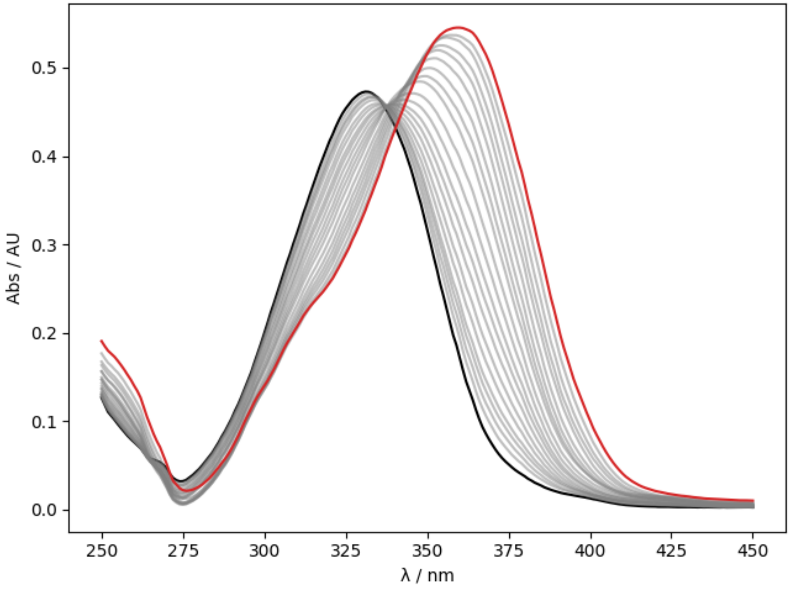

Figure S.36 – UV-vis absorption spectra for the titration of Bu_3PO into **6** (0.0472 mM in *n*-octane, at 298 K). The UV-vis spectrum of the host **6** and the final point of the titration are reported in black and in red, respectively.

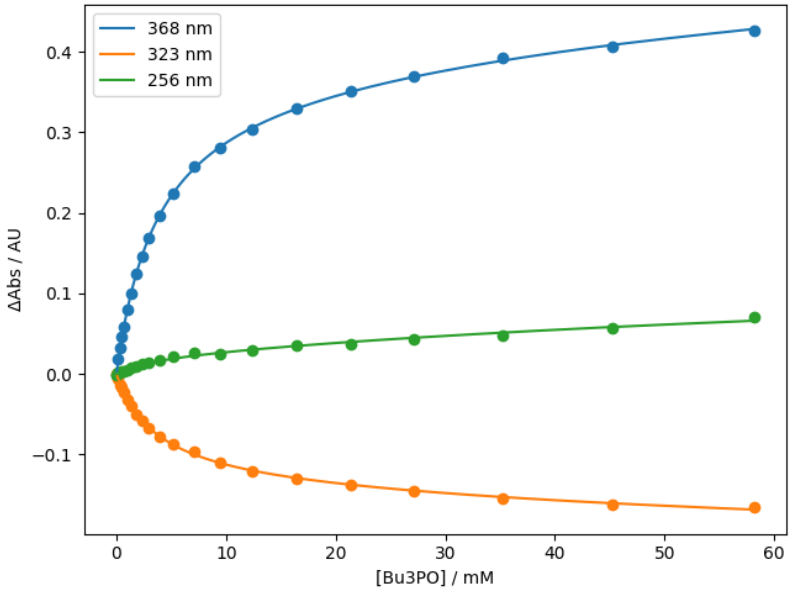

Figure S.37 – The fit of the absorbance at selected wavelengths to a 1:2 binding with a guest absorption isotherm for the titration of Bu_3PO into **6** (0.0472 mM in *n*-octane, at 298 K).

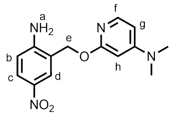

Figure S.38 – Structure of the host **7** with protons labelled for NMR assignment.

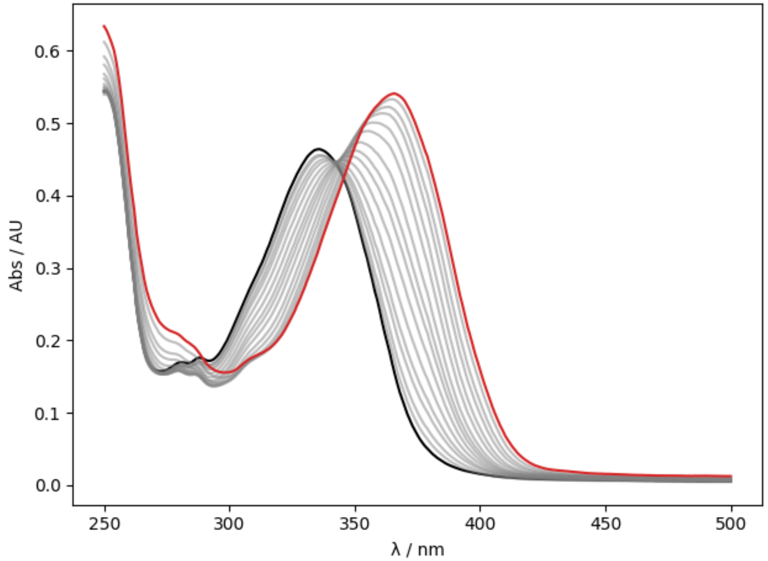

Figure S.39 – UV-vis absorption spectra for the titration of Bu_3PO into **7** (0.0458 mM in *n*-octane, at 298 K). The UV-vis spectrum of the host **7** and the final point of the titration are reported in black and in red, respectively.

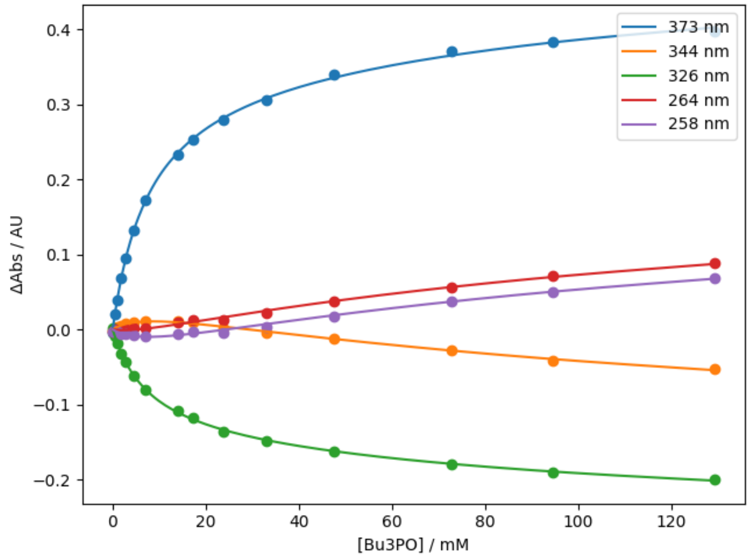

Figure S.40 – The fit of the absorbance at selected wavelengths to a 1:2 binding isotherm for the titration of Bu_3PO into **7** (0.0458 mM in *n*-octane, at 298 K).

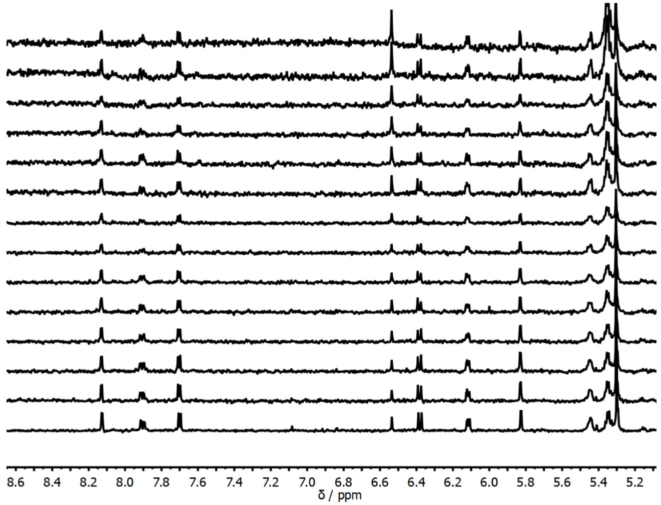

Figure S.41 – Stack plot for the 500 MHz ^1H NMR dilution of **7** in *n*-octane at 298 K ranging from 0.105 mM (bottom) to 0.0243 mM (top). Quartet at 5.34 and singlet at 6.53 are impurities in the solvent.

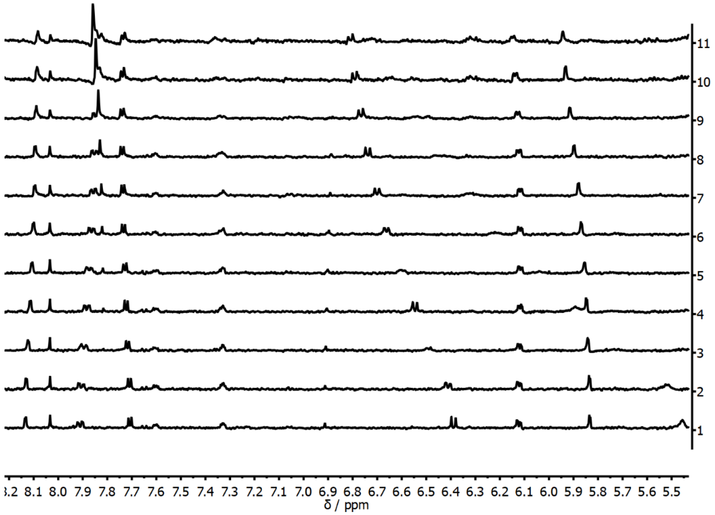

Figure S.42 – Stack plot for the NMR titration of Bu_3PO into **7** (0.468 mM) in *n*-octane at 298 K.

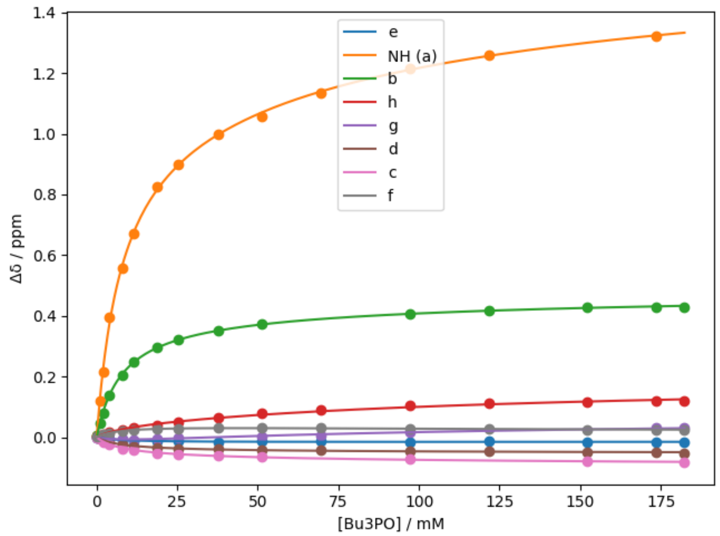

Figure S.43 – Fit of the chemical shift changes to a 1:2 binding isotherm for the NMR titration of Bu_3PO into **7** (2.71 mM) in *n*-octane at 298 K.

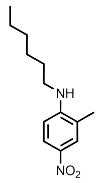

Figure S.44– Structure of the host **8**.

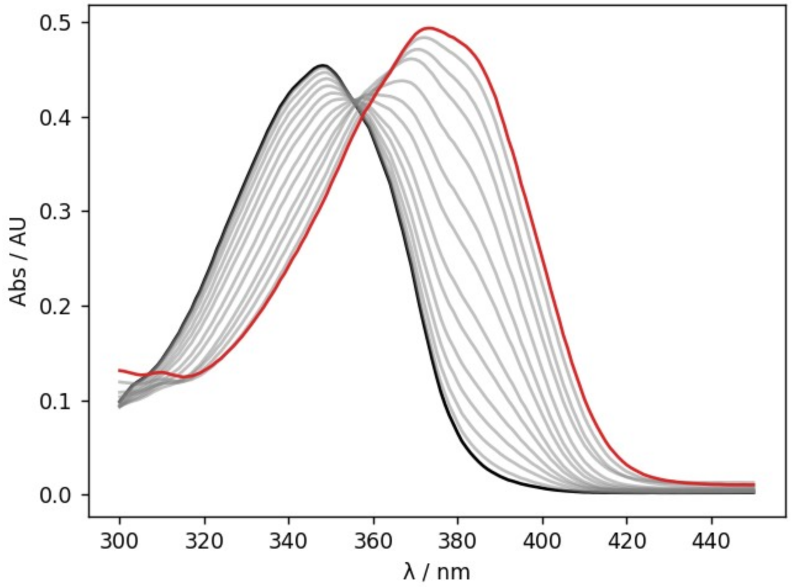

Figure S.45 – UV-vis absorption spectra for the titration of Bu_3PO into **8** (0.0248 mM in *n*-octane, at 298 K). The UV-vis spectrum of the host **8** and the final point of the titration are reported in black and in red, respectively.

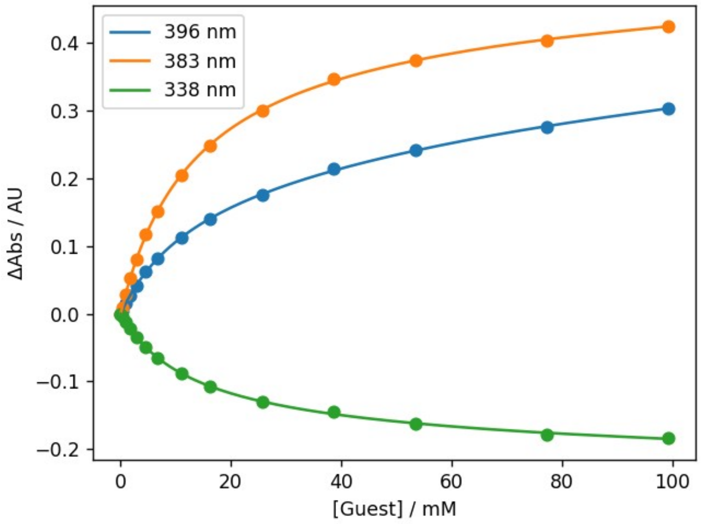

Figure S.46 – The fit of the absorbance at selected wavelengths to a 1:2 binding isotherm for the titration of Bu_3PO into **8** (0.0248 mM in *n*-octane, at 298 K).

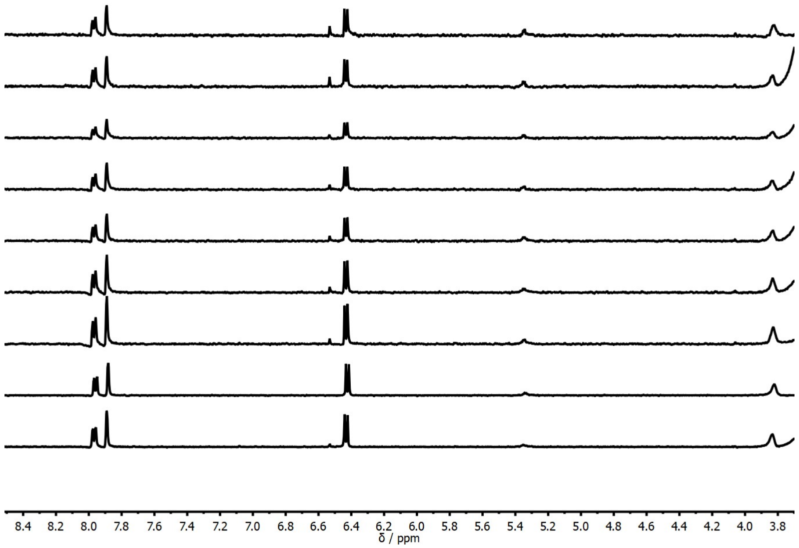

Figure S.47 – Stack plot for the 500 MHz ^{1}H NMR dilution of **8** in *n*-octane at 298 K ranging from 0.825 mM (bottom) to 0.108 mM (top).

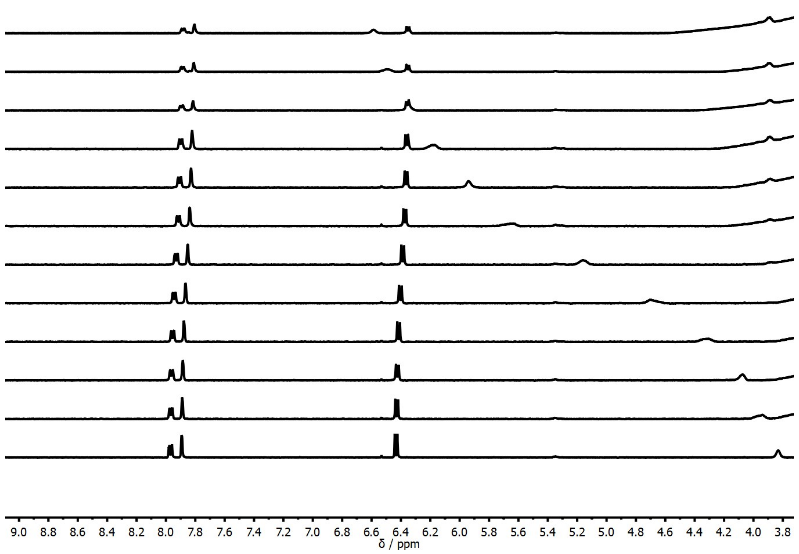

Figure S.48 – Stack plot for the 500 MHz ^{1}H NMR titration of Bu_3PO into **8** (0.825 mM) in *n*-octane at 298 K.

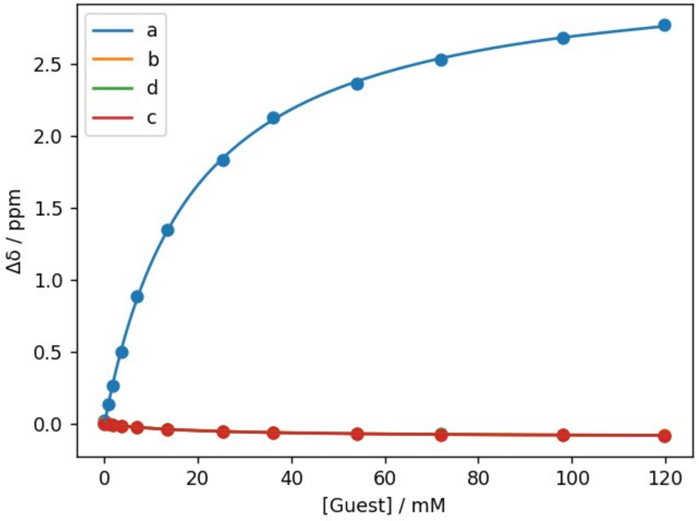

Figure S.49 – Fit of the chemical shift changes to a 1:1 binding isotherm for the NMR titration of Bu_3PO into **8** (0.825 mM) in *n*-octane at 298 K.

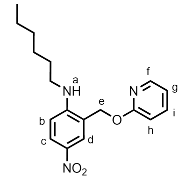

Figure S.50 – Structure of the host **9**.

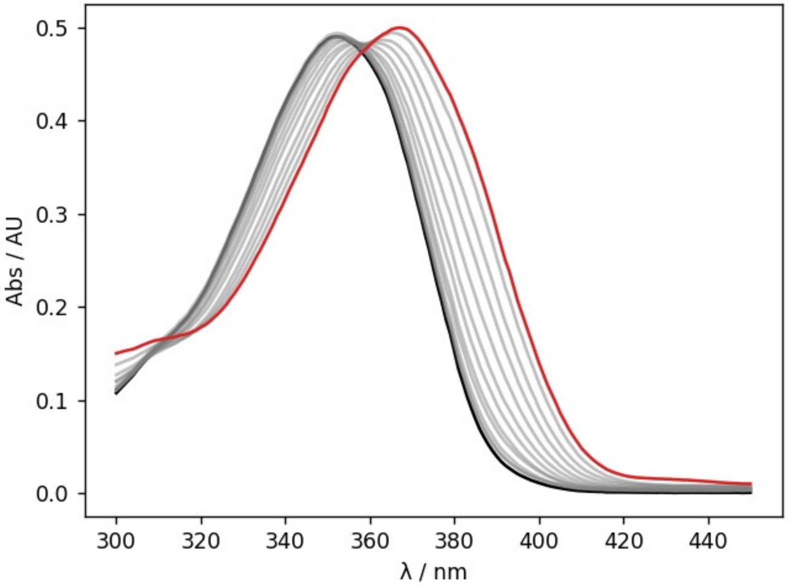

Figure S.51 – UV-vis absorption spectra for the titration of Bu_3PO into **9** (0.0253 mM in *n*-octane, at 298 K). The UV-vis spectrum of the host **9** and the final point of the titration are reported in black and in red, respectively.

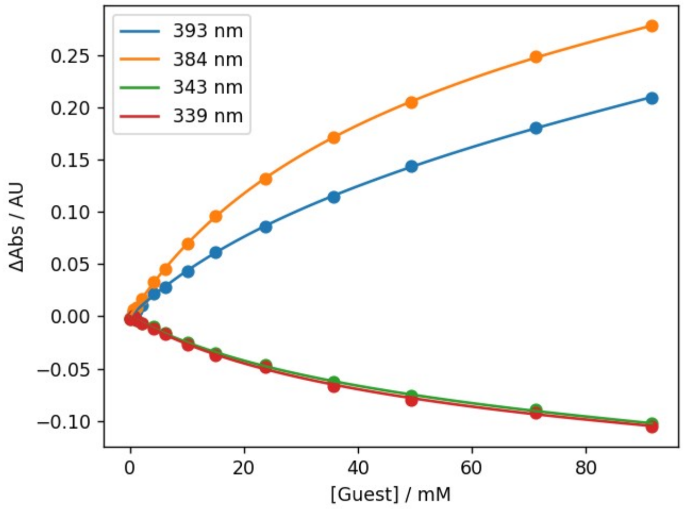

Figure S.52 – The fit of the absorbance at selected wavelengths to a 1:2 binding isotherm for the titration of Bu_3PO into **9** (0.0503 mM in *n*-octane, at 298 K).

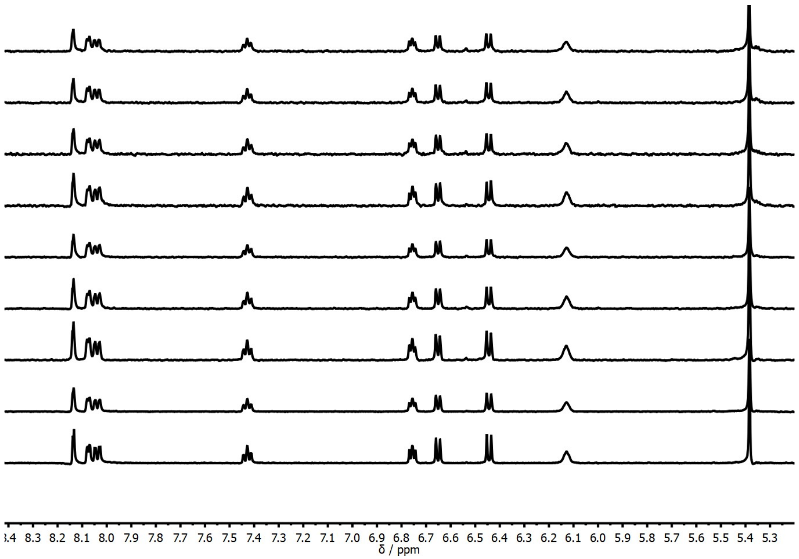

Figure S.53 – Stack plot for the 500 MHz ^{1}H NMR dilution of **9** in *n*-octane at 298 K ranging from 0.844 mM (bottom) to 0.110 mM (top).

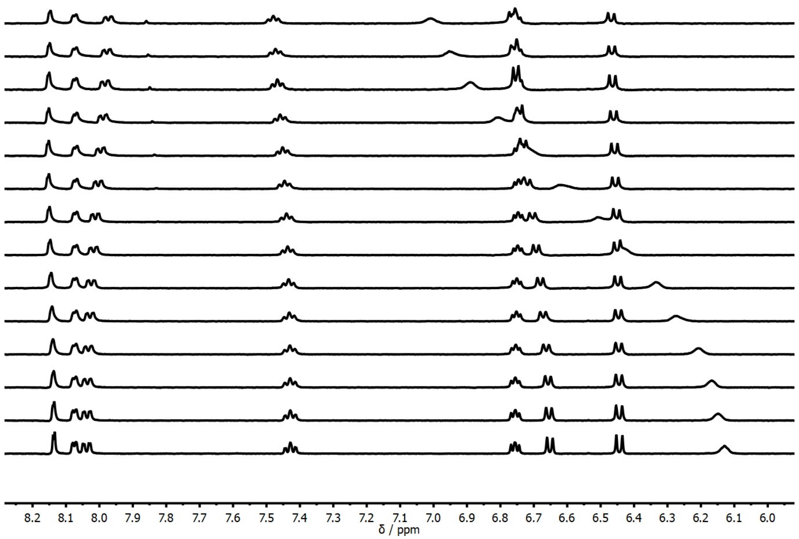

Figure S.54 – Stack plot for the 500 MHz ^{1}H NMR titration of Bu_3PO into **9** (0.844 mM) in *n*-octane at 298 K.

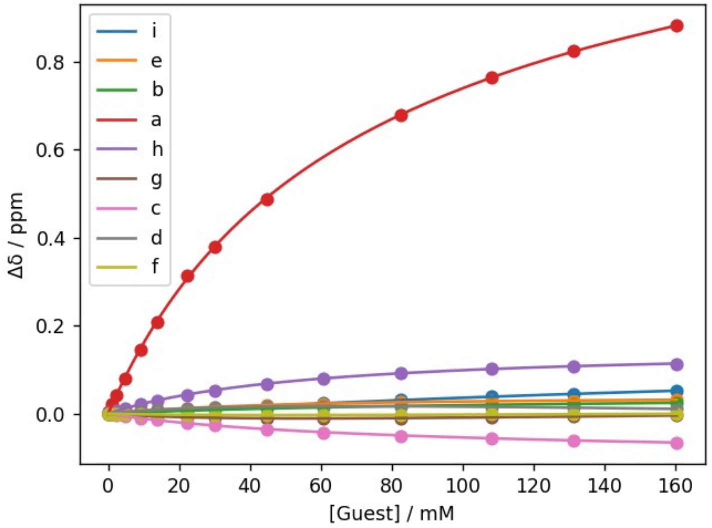

Figure S.55 – Fit of the chemical shift changes to a 1:2 binding isotherm for the NMR titration of Bu_3PO into **9** (0.844 mM) in *n*-octane at 298 K.

Results from fitting of titration data

UV-vis absorption titrations

Compound	X	K_1 / M^{-1}	Maximum percentage of H·nBu$_3$PO in solution	K_2 / M^{-1}	Maximum percentage H·(nBu$_3$PO)$_2$ in solution
**2**	CN	820 ± 30	75	10 ± 11	55
**3**	CF_3	586 ± 14	80	9 ± 2	35
**4**	H	272 ± 11	70	7 ± 5	25
**5**	Me	240 ± 50	70	4 ± 2	50
**6**	OMe	290 ± 2	80	5 ± 2	20
**7**	NMe_2	134 ± 3	75	3 ± 3	25

Table S.3 – Association constants of for the 1:1 and 1:2 complexes of numbered compounds with Bu_3PO as guest and the maximum percentage of host that was present as a given 1:n complex during the course of the titration.

NMR titrations

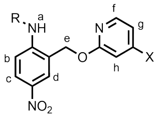

Figure S.56 - Protons labelled for the change in chemical shift in the free species and in the species bound to Bu_3PO. R = H or *n*-hexyl

Compound	X	R	K_1 / M^{-1}	Δδ(a)	Δδ(b)	Δδ(c)	Δδ(d)	Δδ(e)	Δδ(f)	Δδ(g)	Δδ(h)
**1**	N/A	H	320	+1.80	+0.20	-0.09	-0.08	-0.03	-	-	-
**4**	H	H	300	+1.33	+0.36	-0.07	-0.06	-0.03	+0.02	-0.04	+0.00
**7**	NMe_2	H	140	+1.03	+0.38	-0.05	-0.08	-0.01	-0.01	+0.05	+0.05
**8**	N/A	*n*-hexyl	60	+3.18	-0.07	-0.10	-0.09	+0.10	-	-	-
**9**	H	*n*-hexyl	20	+0.94	+0.02	-0.07	+0.05	+0.04	-0.01	-0.03	+0.14

Table S.4 – A table of the shift change for the protons above in Figure S.57 from the free species to the limiting shift upon 1:1 complexation with Bu_3PO. Protons e, f, g and h don't exist in species **1** and **8** as the pyridyloxy group is replaced by a methyl group.

Compound	X	R	K_2 / M^{-1}	Δδ(a)	Δδ(b)	Δδ(c)	Δδ(d)	Δδ(e)	Δδ(f)	Δδ(g)	Δδ(h)
**1**	N/A	H	6	+1.47	+0.07	-0.05	-0.08	+0.08	-	-	-
**4**	H	H	3	+1.32	+0.15	-0.06	-0.04	+0.06	+0.01	+0.05	+0.23
**7**	NMe_2	H	6	+0.59	+0.10	-0.03	-0.01	+0.00	-0.03	+0.09	+0.16
**9**	H	*n*-hexyl	2	+0.50	+0.03	-0.05	-0.11	+0.00	+0.03	+0.09	+0.01

Table S.5 – A table of the shift change for the protons above in Figure S.57 from the limiting shift upon 1:1 complexation to the limiting shift upon 1:2 complexation with Bu_3PO. Protons f, g and h don't exist in species **1** as the pyridyloxy group is replaced by a methyl group. Compound **8** not included as it only shows 1:1 complexation in NMR titrations.

DFT calculations

The crystal structure of **9** was used as the starting point for the calculations for **2** to **7**, with the alkyl chain replaced with a proton and the X group on the pyridine ring changed accordingly. The resulting structures were then used as a basis for DFT optimisation of the newly introduced group with the other coordinates from the crystal structure constrained. The DFT was using a 6-31G* basis set,[5-7] a B3LYP functional[8-11] and a nonrelativistic Hamiltonian (gas phase). The results were used to calculate the molecular electrostatic potential (MEP) on the 0.0104 e bohr^{-3} electron density isosurface in NWChem 7. The MEP was then converted to atomic interaction point (AIP) H-bond parameters using purpose-built Python and JavaScript programmes.[12]

Number	X	Calculated α(aniline)	Experimental α(aniline)	β(pyridine)
**2**	CN	3.3	3.4	5.4
**3**	CF_3	3.2	3.4	5.8
**4**	H	3.0	3.2	7.2
**5**	Me	3.0	3.1	7.7
**6**	OMe	3.0	3.2	7.8
**7**	NMe_2	2.8	3.0	9.5

Table S.6 – Comparison of the H-bond donor parameters for the anilines **2**-**7** calculated using DFT and those measured using UV-vis absorption titrations.

References

1. Fulmer, G.R., Miller, A.J.M., Sherden, N.H., Gottlieb, H.E., Nudelman, A., Stoltz, B.M., Bercaw, J.E., and Goldberg, K.I., *Organometallics,* **2010**, 29, 2176-2179
2. Hanna, F.E., Root, A.J., Hunter, C.A., *Chem. Sci.*, **2023**, 14, 11151-11157
3. Sheldrick, G.M., *Acta Cryst. Sect. A*, **2015**, 71, 3-8
4. Sheldrick, G.M., *Acta Cryst. Sect. C*, **2015**, 71, 3-8
5. Ditchfield, R., Hehre, W. J., Pople, J. A., *J. Chem. Phys.*, **1971**, 54, 724-728
6. Hehre, W. J., Ditchfield, R., Pople, J. A., *J. Chem. Phys.*, **1972**, 56, 2257-2261
7. Hariharan, P.C., Pople, J.A., *Theor. Chim. Acta.*, **1973**, 28, 213-222
8. Becke, A.D., *J. Chem. Phys.*, **1993**, 98, 5648-5652
9. Lee, C., Yang, W., Parr, R.G., *Phys. Rev. B*, **1988**, 37, 785-789
10. Vosko, S.H., Wilk, L., Nusair, M., *Can. J. Phys.*, **1980**, 58, 1200-1211
11. Stephens, P.J., Devlin, F.J., Chabalowski, C.F., Frisch, M.J., *J. Phys. Chem.*, **1994**, 98, 11623-11627
12. Storer, M.C., Zator, K.J., Reynolds, D.P., Hunter, C.A., *Chem. Sci.*, 2023, **15**, 160
